# The mechanism of activation of MEK1 by B-Raf and KSR1

**DOI:** 10.1007/s00018-022-04296-0

**Published:** 2022-05-04

**Authors:** Ryan C. Maloney, Mingzhen Zhang, Yonglan Liu, Hyunbum Jang, Ruth Nussinov

**Affiliations:** 1 Cancer Innovation Laboratory, National Cancer Institute, Frederick, MD 21702 USA; 2 Computational Structural Biology Section, Frederick National Laboratory for Cancer Research, Frederick, MD 21702 USA; 3 Department of Human Molecular Genetics and Biochemistry, Sackler School of Medicine, Tel Aviv University, 69978 Tel Aviv, Israel

**Keywords:** MAPK, KSR, Cancer, ERK, MD simulations, Autoinhibition, Assemblies

## Abstract

MEK1 interactions with B-Raf and KSR1 are key steps in Ras/Raf/MEK/ERK signaling. Despite this, vital mechanistic details of how these execute signal transduction are still enigmatic. Among these is why, despite B-Raf and KSR1 kinase domains similarity, the B-Raf/MEK1 and KSR1/MEK1 complexes have distinct contributions to MEK1 activation, and broadly, what is KSR1’s role. Our molecular dynamics simulations clarify these still unresolved ambiguities. Our results reveal that the proline-rich (P-rich) loop of MEK1 plays a decisive role in MEK1 activation loop (A-loop) phosphorylation. In the inactive B-Raf/MEK1 heterodimer, the collapsed A-loop of B-Raf interacts with the P-rich loop and A-loop of MEK1, minimizing MEK1 A-loop fluctuation and preventing it from phosphorylation. In the active B-Raf/MEK1 heterodimer, the P-rich loop moves in concert with the A-loop of B-Raf as it extends. This reduces the number of residues interacting with MEK1 A-loop, allowing increased A-loop fluctuation, and bringing Ser222 closer to ATP for phosphorylation. B-Raf αG-helix Arg662 promotes MEK1 activation by orienting Ser218 towards ATP. In KSR1/MEK1, the KSR1 αG-helix has Ala826 in place of B-Raf Arg662. This difference results in much fewer interactions between KSR1 αG-helix and MEK1 A-loop, thus a more flexible A-loop. We postulate that if KSR1 were to adopt an active configuration with an extended A-loop as seen in other protein kinases, then the MEK1 P-rich loop would extend in a similar manner, as seen in the active B-Raf/MEK1 heterodimer. This would result in highly flexible MEK1 A-loop, and KSR1 functioning as an active, B-Raf-like, kinase.

**Supplementary Information:**

The online version contains supplementary material available at 10.1007/s00018-022-04296-0.

## Introduction

As the scientific community’s understanding of protein signaling pathways grows, there has been a transition from identifying and describing individual proteins along a pathway towards dimers, nanoclusters, and complexes that allow signals to propagate through dynamic conformational changes and productive high-affinity recruitment [1]. The mitogen-activated protein kinase (MAPK) pathway is one of the most studied signaling pathways due to its role in cell proliferation and tumorigenesis [2–4]. This has led to the development of small-molecule inhibitors that target nearly every step in the MAPK pathway [5, 6]. Despite this progress, drug resistance arises through other, pre-existing or emerging, mutations, paradoxical activation, negative feedback, and activation of alternative pathways [7, 8]. One of the methods currently being investigated to overcome drug resistance is through the development of inhibitors that either prevent complex formation [9] or lock protein complexes in an inactive state [10–12]. Simulations of these complexes can describe how dimerization/complex formation influences protein dynamics, and promotes conformational changes. They can also provide the activation mechanism and insight for drug development.

Dimerization of Raf and the ensuing cascading phosphorylation events are key to the MAPK pathway [13, 14]. Clustering of GTP-bound Ras recruits Raf family proteins to the membrane, increasing their local concentration. The high-affinity interaction of Raf with active Ras shifts the equilibrium from a populated autoinhibited state toward the active state [15, 16], where Raf assumes a side-by-side dimerization of its kinase domain [17]. Active Raf dimer phosphorylates and activates mitogen-activated protein kinase kinase (MEK) in a transient MEK/Raf/Raf/MEK quaternary complex [18, 19]. Exactly how the quaternary complex forms is still unclear. Depending on the relative populations and the affinities of the interactions, multiple routes are possible. Inactive Raf and MEK are known to form face-to-face heterodimers in the cytosol, and two of these can assemble once Raf autoinhibition is relieved [20]. Recent work has shown that MEK and Raf family proteins interact only weakly at basal levels and the epidermal growth factor (EGF) strongly promotes the Raf/MEK interaction, suggesting that Raf dimerization is favored to precede the Raf/MEK interaction [21]. Once Raf has been activated it can phosphorylate MEK [20, 22], or promote a face-to-face MEK homodimerization [23] and autophosphorylation [24]. A MEK dimer then activates extracellular signal-regulated kinase (ERK) [24]. Activated ERK is transported to the cell nucleus where it activates transcription factors, leading to cell proliferation, survival, and growth [25]. Additional proteins (including Hsp90/Cdc37 chaperone complex, 14-3-3 proteins, and kinase suppressor of Ras (KSR)) are integral to proper protein folding, complex stabilization, and allosteric activation along the MAPK pathway [26]. KSR is particularly interesting due to its ability to interact with Raf, MEK, and ERK [27]. Yet, to date its role has been a matter of contention.

The Raf family of serine/threonine kinases consists of A-Raf, B-Raf, and C-Raf (Raf-1) [28]. Each consists of three conserved regions, CR1, CR2, and CR3 (Fig. S1). CR1 is further divided into a Ras-binding domain (RBD) and cysteine-rich domain (CRD). CR2 is a loop that contains a phosphorylation site that can interact with 14-3-3 proteins to stabilize an inactive form of monomeric Raf. CR3 is the kinase domain [29]. In inactive Raf, CR1 and CR3 interact to form an autoinhibited state [30, 31]. Active Ras recruits Raf to the membrane through interactions with the RBD [14, 32] while the CRD binds to the membrane [33–35]. This relieves Raf autoinhibition and allows for dimerization and activation. Of the three isoforms, B-Raf has the highest activity [18, 36] and is frequently mutated in cancer.

MEK1 and MEK2 are dual-specificity threonine/tyrosine kinases and the only known activators of ERK1 and ERK2 [37]. They contain one conserved region, the kinase domain (Fig. S1). N-terminal to the kinase domain is an ERK recognition sequence, a nuclear export sequence, and an autoinhibitory helix [38]. The Raf family of kinases are the best studied MEK activators; however, MEK can also be activated by other MAPK kinases [39–43]. Therefore, MEK can be considered a “gate keeper kinase,” processing signals from multiple upstream activators to control ERK activation. MEK inhibitors (MEKi) have been developed to block signaling in cancers driven by mutations in Ras and Raf. These MEKi, however, can lead to increased signaling through parallel pathways controlled by non-Raf MEK activators, including the c-Jun N-terminal kinase (JNK), p38, ERK5, and nuclear factor-κB (NF-κB) pathways [44].

The mammalian KSR family consists of KSR1 and KSR2. KSR1 contains five conserved regions, CR1–CR5 (Fig. S1), while KSR2 lacks the CR1 domain. CR1 is a coiled coil sterile α motif (CC-SAM) domain that is involved in KSR1 membrane recruitment. It is also able to bind to an N-terminal B-Raf-specific (BRS) region, a region unique to B-Raf [45]. CR2 is a proline-rich (P-rich) domain. CR3 is a CRD acting in membrane recruitment. CR4 contains an ERK-binding motif. CR5 is a kinase or pseudokinase domain [27]. KSR has been considered an active kinase capable of phosphorylating MEK [46, 47], a scaffolding protein involved bringing Raf, MEK, and ERK together [48], and an allosteric activator of B-Raf [45]. How and when KSR interacts with the MAPK pathway is key to understanding cancer progression and acquired inhibitor resistance.

The kinase domains of B-Raf, KSR1, and MEK1 all exhibit typical kinase structures. They consist of a small N-lobe and larger C-lobe connected by a short hinge. Between these two lobes is an ATP-binding pocket. The N-lobe contains five β-strands and an α-helix, called αC-helix. The C-lobe is mostly made up of α-helices (αD through αI) and contains a catalytic HRD motif and activation loop (A-loop) [49, 50]. A sequence alignment of the kinase domains of B-Raf, MEK1, and KSR1 reveals that they are homologues (Fig. S2). The inactive state is characterized by an “outward” position of the αC-helix and a “collapsed” A-loop. In the active state, the αC-helix moves to an “inward” position and the A-loop is “extended.” The “collapsed” A-loop contains an N-terminal “inhibitory helix” that prevents the inward motion of the αC-helix. Activation involves phosphorylation of A-loop residues, which disrupts this “inhibitory helix” and allows the A-loop to extend [50, 51]. In B-Raf the phosphorylated residues are Thr599 and Ser602 [52] and in MEK1 they are Ser218 and Ser222 [53]. KSR1 is known to undergo autophosphorylation, however, it is not known if any residues of the KSR1 A-loop phosphorylate [47]. In addition to these general features of protein kinases, MEK1 also contains a P-rich loop that contains several serine residues (Ser286, Ser292, and Ser298) that undergo phosphorylation and are involved in regulating MEK1 activation/deactivation [54, 55]. KSR1 has long been considered a pseudokinase due to its limited kinase activity [56]. The low kinase activity of KSR1 is often attributed to synonymous changes in key conserved residues found in typical kinases. These include Arg639 in the β3-strand instead of lysine as in B-Raf, and Lys732 in the catalytic motif instead of arginine as in MEK1 and B-Raf, i.e., HKD instead of HRD.

The ability of MEK1 to form face-to-face heterodimers with both B-Raf and KSR1 is an important MAPK feature. This face-to-face recognition is centered around the C-lobe αG-helix. Mutation of a key residue in any of the three proteins abrogates their ability to form the complex (MEK1 F311S, B-Raf I666R, KSR1 W831R). B-Raf is known to phosphorylate MEK1 through a face-to-face interaction [20, 48]. The face-to-face interaction between MEK1 and KSR1 has been implicated in multiple roles. Evidence suggests that under certain circumstances KSR1 can directly phosphorylate MEK1, acting as a true kinase [47]. The direct phosphorylation of MEK1 by KSR1, however, appears to be a low probability event compared to MEK1 phosphorylation by B-Raf. Instead, the primary role of the KSR1/MEK1 heterodimer is to act as either a scaffold or an allosteric activator. As a scaffold, when in a KSR1/MEK1 heterodimer, KSR1 interacts with B-Raf through a side-to-side interface resulting in a B-Raf/KSR1/MEK1 ternary “scaffolding unit.” MEK1 from this unit is then translocated to an active B-Raf dimer nearby and phosphorylated [48, 57]. As an allosteric activator, MEK1 interacts with KSR1 in the cytoplasm forming a “transactivation unit”. This blocks an autoinhibited KSR1 state and enables a side-to-side heterodimer with a B-Raf monomer that has already been recruited to the membrane by Ras. The stabilized active configuration of B-Raf is able to phosphorylate a second MEK1 kinase (that is, not the MEK1 involved in the transactivation unit) [45].

The interactions between B-Raf, MEK1, and KSR1 in the assembly offer a unique system to explore how dynamic and allosteric effects impact protein complex formation, and to examine the mechanism of protein kinase activation by another kinase. There are several B-Raf/MEK1 and KSR1/MEK1 crystal structures in which the substrate MEK1 A-loop is clearly positioned near the activation site of B-Raf or KSR1, whereas in many other crystal structures the substrate is represented by a short peptide bound to the activating kinase. Therefore, these kinase dimer crystal structures offer an ideal starting point to gain insight into how kinases really interact during phosphorylation. Molecular dynamics (MD) simulations based on these crystal structures can capture dynamic changes around the activation site that can be difficult to visualize in a laboratory setting as these interactions are likely highly transient, with the substrate leaving the activator rapidly in the phosphorylated state.

In this study, we performed MD simulations of active B-Raf/MEK1, inactive B-Raf/MEK1, and KSR1/MEK1 heterodimers. Coupled with the available data, these simulations allow us to investigate (i) *why* B-Raf activation is necessary to phosphorylate MEK1, (ii) *what* occurs at the interface between active B-Raf and MEK1 that leads to phosphorylation, and (iii) *why* B-Raf is more potent at activating MEK1 than KSR1. Our results show that the P-rich loop of MEK1 moves in concert with the B-Raf A-loop which influences the flexibility of the MEK1 A-loop. The collapsed A-loop in inactive B-Raf draws MEK1 P-rich loop towards MEK1 A-loop, reducing the A-loop flexibility. When the B-Raf A-loop is extended, the MEK1 P-rich loop moves with it, repositioning it towards the bottom of the MEK1 C-lobe. Once this has occurred, the MEK1 A-loop becomes more flexible and is able to orient Ser222 towards the ATP in B-Raf. The increased flexibility in the MEK1 A-loop also allows B-Raf αG-helix residue Arg662 to move from a position within the A-loop of MEK1 to a position outside of the A-loop “inhibitory helix.” The motion of Arg662 allows the MEK1 A-loop to reorient, bringing Ser218 closer to ATP. Our results also show that additional residues in KSR1 compared to B-Raf lead to steric clashes at the KSR1/MEK1 interface and result in different dynamics in the two complexes. This creates a large gap between the N-lobes of the two proteins and has implications in KSR1’s ability to function as an active kinase or scaffold.

## Results

### Crystal structure

**Fig. 1:**
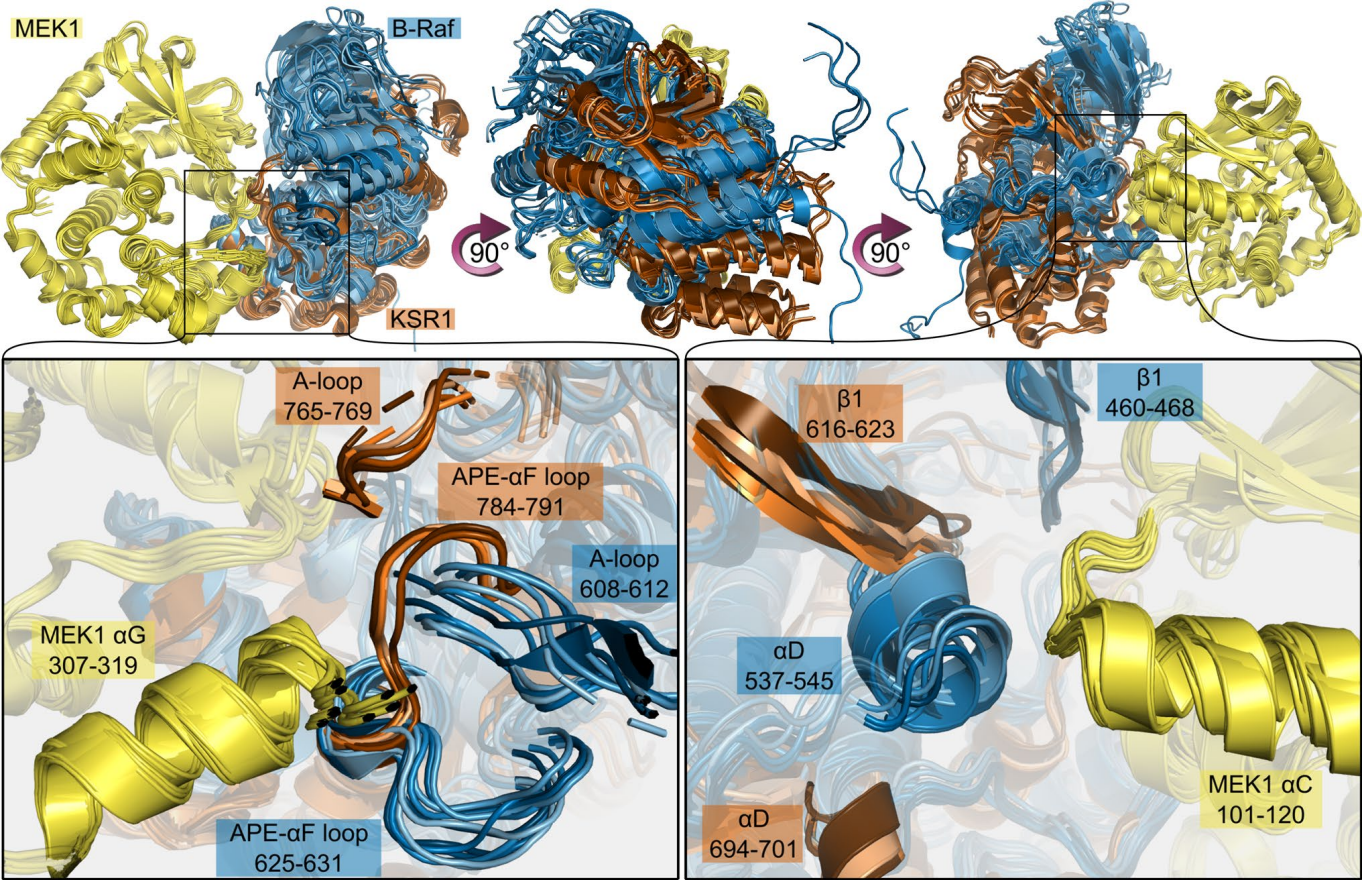
Crystal structures show structural differences in the intermolecular interfaces between B-Raf/MEK1 and KSR1/MEK1. MEK1 is shown in yellow, B-Raf in blue, and KSR1 in orange. PDB IDs for B-Raf/MEK1 structures: 4MNE, 6NYB, 6PP9, 6Q0J, 6Q0T, 6V2W, 7M0T, 7M0U, 7M0V, 7M0W, 7M0Z. PDB IDs for KSR1/MEK1: 7JUW, 7JUX, 7JUY, 7JUZ, 7JV0, 7JV1

As an initial step towards comparing the interfaces of the B-Raf/MEK1 and KSR1/MEK1 heterodimers, we overlaid the available crystal structures of the complexes (Fig. 1). The structures were aligned using Pymol [58], based on the positions of MEK1 C_α_ atoms. From this image, we see significant structural differences between the two complexes. First, the N-lobe of B-Raf is tilted closer to the N-lobe of MEK1 compared to that of KSR1. Second, KSR1 appears to be rotated clockwise relative to the position of B-Raf (see middle image of Fig. 1). Not only are the relative positions of KSR1 and B-Raf different when they interact with MEK1, there are key differences in regions of the proteins that are in contact with each other. The superimposed structures highlighting the position of the A-loop and the APE-to-αF loop of KSR1 and B-Raf relative to the position of MEK1 αG-helix show that MEK1 αG-helix is positioned near B-Raf residues in both the A-loop and APE-to-αF loop (left inset of Fig. 1). In the KSR1/MEK1 heterodimer, however, the αG-helix of MEK1 is only near KSR1 residues of the APE-to-αF loop. KSR1 A-loop residues are positioned away from MEK1 αG-helix. Sequence alignment of B-Raf and KSR1 (Fig. S2) shows that KSR1 has three additional residues in the A-loop and six residues between the A-loop and αF-helix when compared to B-Raf. These additional residues, and the steric interactions resulting from them, could explain why residues from KSR1 A-loop do not interact with residues from MEK1 αG-helix. The superimposed positions of KSR1 and B-Raf relative to the position of MEK1 αC-helix highlight the impact of the clockwise rotation of KSR1 relative to B-Raf (right inset of Fig. 1). It shows that MEK1 αC-helix interacts with B-Raf αD-helix, while KSR1 αD-helix, is shifted down, away from MEK1 αC-helix. This positions the β1-strand of KSR1 near MEK1 αC-helix, while the corresponding B-Raf β1-strand is located closer to the β-strands of MEK1. The differences present in the crystal structures of the two dimeric systems were used to help guide our analysis of the MD simulation results.

### Overall structural movement

**Fig. 2:**
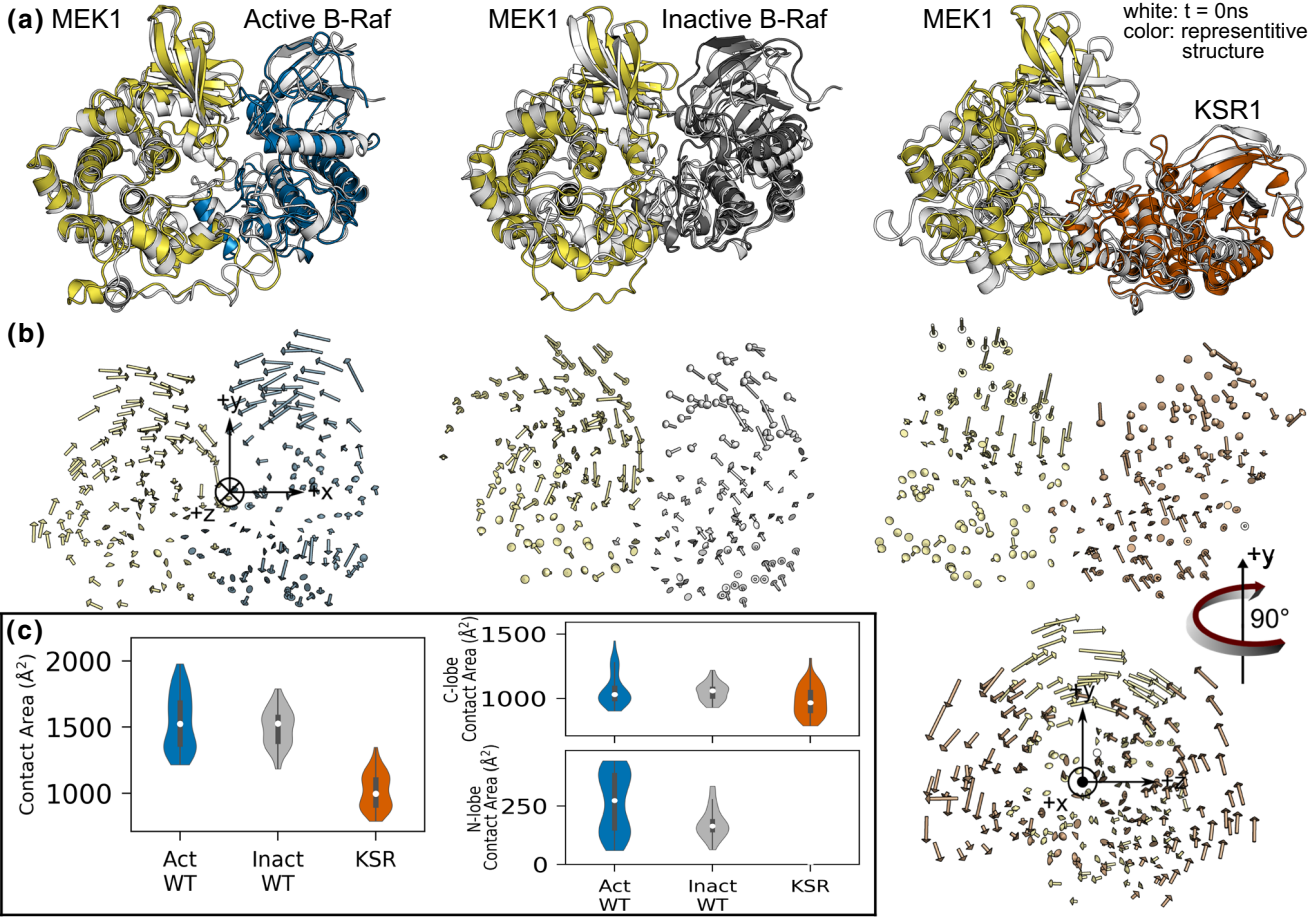
**a** Snapshots comparing the initial structure with the most representative structure for the B-Raf/MEK1 and KSR1/MEK1 heterodimers. The N-lobes in B-Raf and MEK1 move towards each other while the N-lobes of KSR1 and MEK1 move apart. Cartoon drawings of the initial configuration (white) and a representative snapshot of the active wild-type B-Raf/MEK1 (blue/yellow), inactive wild-type B-Raf/MEK1 (gray/yellow) and KSR1/MEK1 (orange/yellow) systems. **b** First normal mode of every other residue for the same systems as above. **c** Total contact area, C-lobe to C-lobe contact area, and N-lobe to N-lobe contact area for active wild-type B-Raf/MEK1 (Act WT), inactive wild-type B-Raf/MEK1 (Inact WT) and KSR1/MEK1 (KSR) systems

The differences between the B-Raf/MEK1 and KSR1/MEK1 heterodimers seen in the crystal structures are highlighted in the overall structural movement seen in our simulations. We compared the initial structure with the most representative structure for the B-Raf/MEK1 and KSR1/MEK1 simulations (Fig. 2a) and superimposed representative snapshots for the top five most populated configuration subfamilies (Fig. S3). The N-lobes of B-Raf and MEK1 move closer towards each other, while the N-lobes of KSR1 and MEK1 move away from each other. This observation is confirmed through principal component analysis (PCA) on each simulation trajectory. In the PCA calculations, we have excluded residues from the A-loop of B-Raf and KSR1, as well as residues from the P-rich loop in MEK1. This was done to highlight the motion of the bulk of the protein, which is otherwise overshadowed by the flexibility of these loops (see Fig. S4 for root mean square fluctuation (RMSF) results). The first normal mode from the PCA calculations (Fig. 2b, Supplementary movies) for active wild-type B-Raf/MEK1, inactive wild-type B-Raf/MEK1, and KSR1/MEK1 systems indicates that the primary motion of both B-Raf systems has B-Raf rotating counterclockwise and MEK1 rotating clockwise to bring their respective N-lobes into closer contact (when viewed along the + *z* axis as indicated), in agreement with previous simulations of the B-Raf/MEK1 dimer [21]. KSR1, however, exhibits different behavior for the first normal mode. In this system, the proteins’ N-lobes rotate away from each other, with KSR1 rotating counterclockwise and MEK1 rotating clockwise when viewed along the –*x* axis. We also calculated the first normal mode for other trajectories (Fig. S5) and found the same behaviors as those depicted in Fig. 2b. The variance of the first normal mode (Fig. S6) is between 0.25 and 0.30 for most trajectories, with the second normal mode variance being about 0.15. These values of variance indicate that the broad structural motion described above captures a good portion of the protein motion; however, additional modes are needed to gain a fuller picture of the dynamics. The second and third normal modes (Supplementary movies) show smaller motion in the bulk of the protein than is seen in the first normal mode revealed. Instead, these normal modes highlight motion in other regions such as B-Raf αC-helix and MEK1 A-loop or C-lobe α-helices.

### Comparison of protein–protein contact area

Inspired by the apparent increase in N-lobe contact area seen in the B-Raf/MEK1 simulations, and results reported by previous MD simulations of this system [21], we calculated the protein–protein contact area for the active and inactive wild-type B-Raf/MEK1 systems as well as for the KSR1/MEK1 system (Fig. 2c). Additional calculations were also performed for other systems including B-Raf V600E and pThr599/pSer603 B-Raf with MEK1 (Fig. S7a-c). These results show that the total contact area between B-Raf and MEK1 is greater than that between KSR1 and MEK1. We break down the total contact area to compare just C-to-C-lobe and N-to-N-lobe contact area. This shows that the C-to-C-lobe contact area is approximately equal for all systems, while there is no N-to-N-lobe contact in the KSR1 systems. The greater contact area is reflected in the Gibb’s free energy of binding (Fig. S7d), which shows that the systems with higher contact area have lower values for ${\Delta G}_{\mathrm{binding}}$. The results are less clear when we look at the change in contact area throughout the course of the simulation (Fig. S8). It has previously been reported that the B-Raf/MEK1 contact area increased over the course of 25 µs simulations, and that this increase was greater for active B-Raf/MEK1 systems than for inactive B-Raf/MEK1 systems [21]. Our results indicate that the total contact area does not follow a consistent trend, so we cannot draw a definitive conclusion as to whether the proteins move closer together based on contact area alone. For some simulations, N-to-N-lobe contact area does appear to be increasing; however, this is often offset by a decrease in C-to-C-lobe contact area so that the net result is an approximately constant total contact area.

Hydrogen bonds (H-bond) and salt bridges can stabilize protein–protein interactions and contribute to binding specificity, and/or dissociation. We plot the interfacial H-bond and salt-bridge contact area in Fig. S9. The H-bond contact area is approximately equal across all investigated systems when both the N- and C-lobes are considered. However, if only the C-lobe is considered, the KSR1/MEK1 system generally displays more hydrogen bonding than the B-Raf/MEK systems. The results for salt-bridge contact area show that B-Raf creates more electrostatic interactions with MEK1 than KSR1 does (when considering both lobes), and that active B-Raf creates more than inactive B-Raf. In the C-lobe, the inactive wild-type and KSR1 systems have comparable salt-bridge interactions. The active B-Raf systems display a bimodal distribution. One peak is approximately equal to those of the inactive wild-type and KSR1 systems, and the second peak is higher. Structures in the active systems that exhibit higher values of salt-bridge interactions display two salt bridges that are not formed in any of the inactive B-Raf/MEK1 systems. The first salt bridge involves B-Raf Arg662 (at the N-terminal end of the αG-helix) and MEK1 Asp217 (in the MEK1 A-loop, adjacent to Ser218). The second salt bridge is between B-Raf Asp663 (at the N-terminal end of the αG-helix) and MEK1 Arg189 (the aspartic acid in MEK1 HRD motif). The impact of the salt bridge between Arg662 and Asp217 is explored further in “B-Raf (or KSR1) αG-helix to MEK1 A-loop”.

### Structural changes of B-Raf and KSR1

Kinases are typically classified as being in an active or inactive configuration based on the position of the αC-helix and the orientation of the DFG motif. Following the technique outlined in our previous work [59], we define the position of the αC-helix as “in” or “out” based on the formation of a salt bridge between B-Raf Lys483 on β3-strand and Glu501 on αC-helix. We define the salt bridge as formed if the distance between the C_β_ atom on the sidechain of Lys483 and the C_β_ atom on the sidechain of Glu501 is less than 10 Å (Fig. S10a). Since KSR1 lacks a lysine residue on the β3-strand, we measure the distance between the C_β_ atom of Arg639 (on the β3-strand) and the C_β_ atom on the sidechain of Glu657 (on the αC-helix), applying the same distance criterion. The results indicate that systems that begin with an “out” αC-helix maintain this orientation throughout the simulations (inactive wild-type B-Raf and KSR1). Systems that begin with an “in” αC-helix (active wild-type B-Raf, phosphorylated wild-type B-Raf, and B-Raf V600E) largely maintain this orientation; however, there are some points at which the salt-bridge breaks, particularly for the active wild-type B-Raf systems.

To describe the orientation of the DFG motif, we adopt the technique proposed by Modi and Dunbrack [60]. In their work, they show that nearly all active kinases exhibit a specific dihedral conformation for the XDFG motif, where X refers to the residue at the −1 position relative to the DFG motif. This conformation, called “BLAminus” indicates that the X residue occupies the β (B) region of the Ramachandran map, the DFG aspartic acid occupies the left (L) region, and the DFG phenylalanine backbone occupies the α (A) region, while its’ first rotamer (χ_1_) adopts a gauche-minus (−60°) orientation. Measurements of these dihedral angles indicate that inactive systems do not satisfy the criteria for an active kinase, with the DFG-Phe backbone primary in the β region of Ramachandran map, and its sidechain exhibiting gauche-plus (+60°) and gauche-minus angles (Fig. S11a). Systems that began with an active configuration generally maintain the “BLAminus” XDFG motif, however, the active wild-type and phosphorylated wild-type simulations do show some deviation from this orientation. Specifically, the X residue moves away from the β region towards the left region, while the DFG aspartic acid does the reverse. In addition, the χ_1_ rotamer adopts a gauche-plus orientation for the phosphorylated B-Raf simulation. This indicates that the B-Raf kinase domain does not maintain an active orientation throughout the simulation which could be expected because full activation of wild-type B-Raf requires B-Raf side-to-side dimerization with a second Raf kinase or KSR. B-Raf V600E, however, does not require side-to-side dimerization to maintain an active conformation, and results indicate that it maintains “BLAminus” configuration for the XDFG motif.

### Structural changes of MEK1

The initial orientation for every MEK1 protein in our simulations was an inactive orientation with an “out” αC-helix. We determined the position of the αC-helix in MEK1 as we did for B-Raf and KSR1, using the distance between the sidechain C_β_ atoms of Lys97 and Glu114 as basis for our measurement. The αC-helix maintains an outward position throughout the simulation (Fig. S10b). Likewise, results of the XDFG dihedrals show that the DFG motif never obtains the active “BLAminus” configuration (Fig. S11b).

**Fig. 3:**
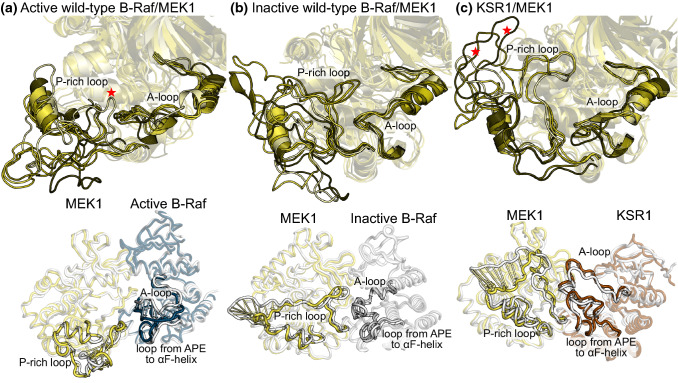
MEK1 P-rich loop adopts different configurations based on if it interacts with active B-Raf, inactive B-Raf, or KSR1. Representative structures for the five most populated configuration subfamilies of the ensemble trajectories for **a** the active wild-type B-Raf/MEK1, **b** inactive wild-type B-Raf/MEK1, and **c** KSR1/MEK1 systems (top row). The first normal mode motion of the MEK1 P-rich loop and B-Raf (or KSR1) A-loop (up to the αF-helix) is shown in the bottom row

The area of MEK1 that does exhibit large structural changes is the P-rich loop. We observed that in the active wild-type B-Raf/MEK1 system this loop is collapsed near the bottom of the C-lobe, and away from the MEK1 A-loop for all representative configurations except for one, which is marked with a red star (Fig. 3a). The first normal mode of the P-rich loop shows that this loop moves in conjunction with the B-Raf A-loop towards the loop between the APE motif and αF-helix (Supplemental movies). The P-rich loop behaves in a similar manner for the other active B-Raf systems (Fig. S12). The P-rich loop of MEK1 for inactive B-Raf/MEK1 systems has significantly more contacts with MEK1 A-loop than it did in active B-Raf systems (Fig. 3b). The first normal mode again shows that the MEK1 P-rich loop is positioned to interact with the A-loop of B-Raf. In the KSR1/MEK1 system, the P-rich loop either extends backwards to interact with the αI-helix (marked with red stars) or upwards to interact with MEK1 A-loop and N-lobe (Fig. 3c). When the P-rich loop extends upwards, it is positioned to interact with the A-loop and the loop between the APE motif and the αF-helix. The relocation of the MEK1 P-rich loop when MEK1 interacts with the active B-Raf compared to inactive B-Raf or KSR1 has not been reported previously.

The position of the P-rich loop either adjacent to or separated from the A-loop of MEK1 could have a key role in the activation of MEK1. To decipher how the conformational dynamics of these loops affect the activation of MEK1, we calculated the RMSF of MEK1 A-loop for the active/inactive wild-type B-Raf/MEK1 and KSR1/MEK1 systems (Fig. S13a). In inactive wild-type B-Raf/MEK1 the RMSF values are lower than those of active wild-type B-Raf/MEK1. Results for the other systems including phosphorylated B-Raf and B-Raf V600E follow the same trend (Fig. S13b), with RMSF values around Ser218 and Ser222 being higher in the active systems than the inactive wild-type B-Raf/MEK1 system. This could indicate that the MEK1 P-rich loop acts to stabilize MEK1 A-loop when B-Raf is inactive. Activation of B-Raf allows the P-rich loop to move and relieves stabilizing interactions from the MEK1 A-loop, allowing the A-loop to fluctuate. However, this cannot be the case in the KSR1/MEK1 system, where the MEK1 P-rich loop is in a similar position to that of the inactive wild-type B-Raf/MEK1 system yet the MEK1 A-loop maintains large RMSF values relative to the other two systems. The reason for the large RMSF values of MEK1 A-loop in the KSR1/MEK1 system will be explored in “B-Raf (or KSR1) αG-helix to MEK1 A-loop”.

### Interfacial contacts

Up until this point, we have focused on general protein dynamics: discussing how the two proteins in the dimer system move in relation to each other and how each protein behaves individually. From this, we have seen that the proteins in the B-Raf /MEK1 heterodimer move to bring their N-lobes closer to each other, while the proteins in the KSR1/MEK1 heterodimer move to bring the N-lobes away from each other. In addition, we have shown that there were no large-scale changes to individual protein configurations (active proteins remained active and inactive proteins remained inactive). Now, we will focus on the interface between the proteins. To determine how individual residues impact the dimer dynamics, we generated intermolecular contact maps for the B-Raf/MEK1 and KSR1/MEK1 systems (Fig. S14). The high residue–residue contact probability indicates four distinct regions of contacts in each of our systems: B-Raf (or KSR1) αG-helix to MEK1 αG-helix, B-Raf (or KSR1) αG-helix to MEK1 A-loop, B-Raf (or KSR1) A-loop to MEK1 αG-helix, and B-Raf (or KSR1) A-loop to MEK1 A-loop. In addition, there are contacts between the N-lobes of B-Raf and MEK1, but not between KSR1 and MEK1.

#### B-Raf (or KSR1) A-loop to MEK1 αG-helix

**Fig. 4:**
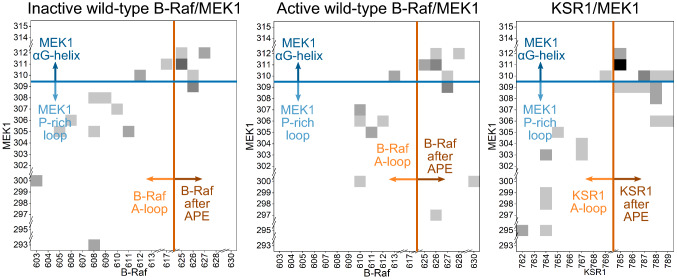
MEK1 P-rich loop makes more contact with inactive B-Raf or KSR1 A-loop residues than with active B-Raf A-loop residues. Contact maps for MEK1 P-rich loop and αG-helix resides versus B-Raf (or KSR1) A-loop residues and the residues between the APE motif and αF-helix for the inactive wild-type B-Raf/MEK1 (*left panel*), active wild-type B-Raf/MEK1 (*middle panel*), and (c) KSR1/MEK1 (*right panel*) systems

In “Structural changes of MEK1” section, we showed that the position of the MEK1 P-rich loop changes based on the position of the A-loop of B-Raf or KSR1. The contact map between the MEK1 P-rich loop/αG-helix and the A-loop up to the αF-helix of KSR1 shows that KSR1 residues after the APE motif make more contact with MEK1 P-rich loop and αG-helix residues than do residues after the APE motif in the B-Raf systems (Fig. 4). This is likely due to the additional six residues between the APE motif and αF-helix in KSR1 compared to B-Raf. The contact map also shows that there are more interactions between MEK1 P-rich loop residues and the A-loop of inactive B-Raf and KSR1 than between MEK1 and active B-Raf, in agreement with our results from the normal mode analysis (Fig. 3). Results from the pThr599/pSer603 and B-Raf V600E follow the same trends (Fig. S15), with very few contacts between B-Raf A-loop and MEK1 P-rich loop when B-Raf is active, and strong contacts between residues in these two loops when B-Raf is inactive.

#### B-Raf (or KSR1) αG-helix to MEK1 αG-helix

The importance of the αG-helix in dimer formation has been well documented, and mutations of key residues in B-Raf, KSR1, and MEK1 can result in disruption of dimer formation [45]. The contact maps between the αG-helices for all systems show that the contacts between B-Raf/MEK1 and KSR1/MEK1 largely match each other (Fig. S16). Differences do arise, however, between the beginning and ending residues of the B-Raf and KSR1 αG-helices that impact how they interact with MEK1. The C-terminal residue of the B-Raf αG-helix, Arg671, has a much longer sidechain than that of the corresponding KSR1 residue, Ser835. This results in more contacts between B-Raf and MEK1 than between KSR1 and MEK1, including a salt-bridge formation between B-Raf Arg671 and MEK1 Asp315. At the N-terminal end of the B-Raf and KSR1 αG-helices, inactive systems exhibit more contacts with MEK1 than with active B-Raf systems. The N-terminal end of the B-Raf and KSR1 αG-helices are positioned near the A-loop of MEK1, so we next explore this region of the dimer interface.

#### B-Raf (or KSR1) αG-helix to MEK1 A-loop

**Fig. 5:**
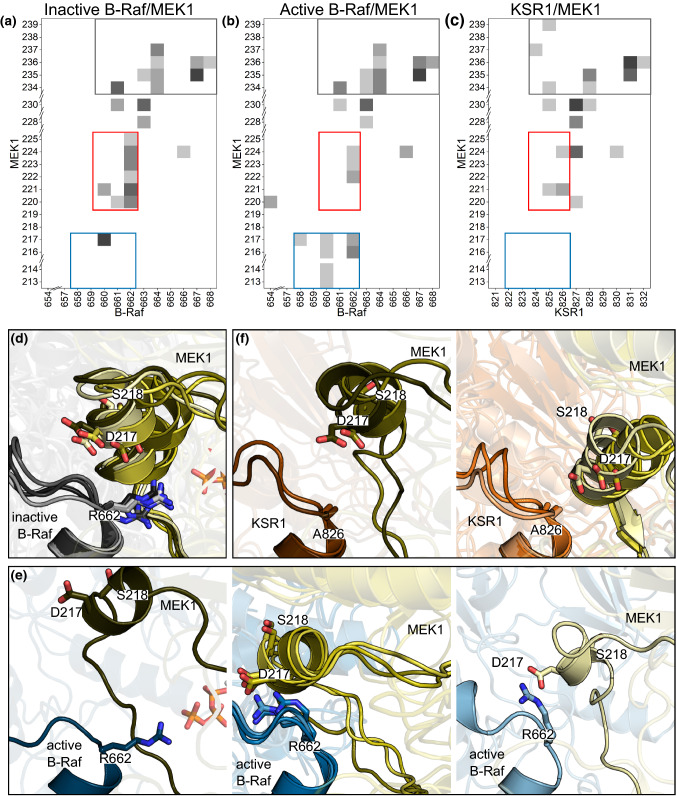
MEK1 A-loop displays different interactions with the αG-helix of inactive B-Raf, active B-Raf, and KSR1. Contact maps for MEK1 A-loop through αF-helix residues versus B-Raf (or KSR1) αG-helix residues including the residues from the N-terminal end of αG-helix for **a** inactive wild-type B-Raf/MEK1, **b** active wild-type B-Raf/MEK1, **c** and KSR1/MEK1 systems. Representative snapshots of **d** inactive B-Raf/MEK1, **e** active B-Raf/MEK1, and **f** KSR1/MEK1 systems, aligned with respect to the B-Raf (or KSR) αG-helix position

The N-terminal ends of B-Raf and KSR1 αG-helices are positioned near Ser218 and Ser222 on MEK1, the two residues that need to be phosphorylated to activate MEK1. As such, the position of the αG-helix could play an important role in positioning MEK1 for phosphorylation. Contact maps between B-Raf (or KSR1) αG-helix and MEK1 A-loop show that in all systems there is significant interaction between residues around the B-Raf (or KSR1) αG-helices and residues in MEK1 just after the SPE motif (residues 231–233) at the end of the A-loop (Fig. 5a–c, gray box). Differences occur, however, with the interactions between B-Raf (or KSR1) and MEK1 residues around these two serine residues. There is more contact between inactive wild-type B-Raf residues 660–662 and MEK1 residues 220–225 (residues around Ser222) than there is between these residues in active wild-type B-Raf/MEK1 or the corresponding residues 824–826 in KSR1/MEK1 (Fig. 5a–c, red box). On the other hand, active wild-type B-Raf residues 658–662 make greater contact with MEK1 residues 213–217 (residues that lie on the A-loop “inhibitory-helix”) than the inactive B-Raf/MEK1 or KSR1/MEK1 systems (Fig. 5a–c, blue box).

Snapshots of these three systems can help to explain the differences seen in the contact maps. Representative snapshots for the top five most populated ensemble clusters shows that Arg662 of inactive wild-type B-Raf is positioned inside the MEK1 A-loop, forming a salt-bridge with the oxygen atoms on the γ-phosphate of ATP (Fig. 5d). However, Arg662 of active wild-type B-Raf (Fig. 5e) can either occupy the same position as seen in the inactive system (inside the MEK1 A-loop, left panel), or it can move outside the inhibitory helix and form a salt bridge with Asp217 on MEK1 (center and right panel). In KSR1, the residue that corresponds to Arg 662 is Ala826. Due to alanine being a shorter, uncharged residue compared to arginine, Ala826 makes much less contact with MEK1 (Fig. 5f). One result of the shorter residue in KSR1 and the movement of Arg662 in active wild-type B-Raf is that the “inhibitory-helix” of MEK1 can move more in these systems than in the inactive wild-type system. This can be seen in the snapshots, which are aligned with respect to the positions of the C_α_ of the B-Raf (or KSR1) αG-helix (Fig. 5d–f). These show that in the inactive wild-type B-Raf system, the C-terminal ends of the “inhibitory-helices” of MEK1 are all in the same position. In active wild-type B-Raf, the MEK1 A-loop “inhibitory-helix” adopts three different positions and orientations. In KSR1, the “inhibitory-helix” adopts 2 different positions.

In Fig. S13, we showed that the RMSF values of MEK1 A-loop residues in the active wild-type B-Raf/MEK1 and KSR1/MEK1 systems were larger than those in the inactive wild-type system. While we attributed the difference in RMSF values for the two B-Raf/MEK1 systems to the impact of the MEK1 P-rich loop, Arg662 could also play a role as seen in Fig. 5. Particularly for the KSR1/MEK1 system, the increased RMSF values can be attributed to the presence Ala in KSR1 as opposed to Arg in B-Raf. Taken together, the position of MEK1 P-rich loop, the movement of Arg662 (or substitution with Ala826 in KSR1), and the increased MEK1 A-loop RMSF begin to reveal important differences between the active B-Raf, inactive B-Raf, and KSR1 systems that may give clues to the structural changes required for MEK1 phosphorylation. Next, we focus on the contact area between the A-loops of B-Raf (or KSR1) and MEK1 to explore this further.

#### B-Raf (or KSR1) A-loop to MEK1 A-loop

**Fig. 6:**
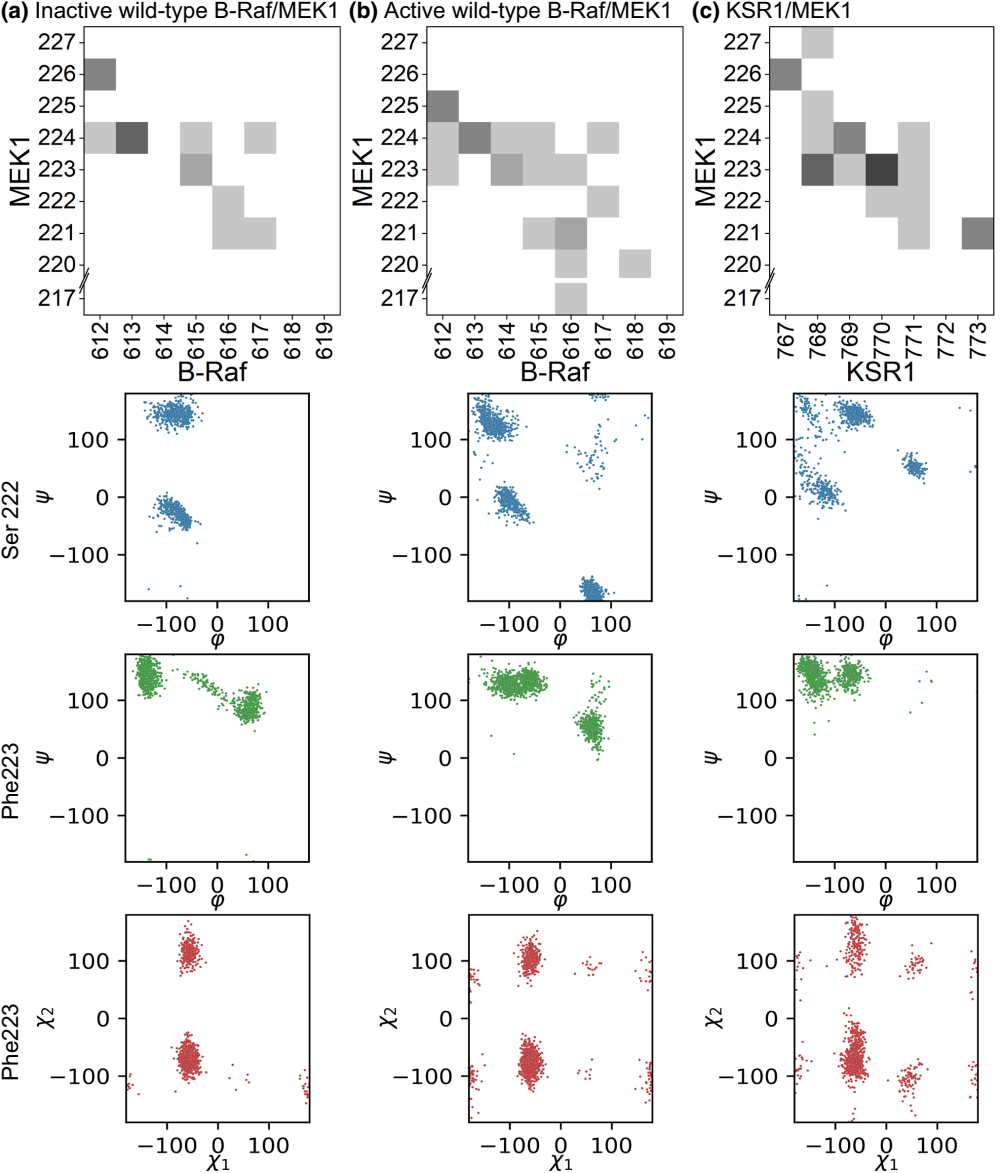
Increased flexibility in the MEK1 A-loop leads to increased rotation of Ser222 and Phe223 and more contact between MEK and B-Raf (or KSR1) A-loop. Contact map (top), main chain dihedral angles of Ser222 (blue), main chain dihedral angle of Phe223 (green), and sidechain dihedral angles of Phe223 (red) of MEK1 for **a** inactive B-Raf/MEK1, **b** active B-Raf/MEK1, and **c** KSR1/MEK1 systems

Steps involved in the phosphorylation of kinase substrates have been described previously; however, most of these descriptions have focused on changes to the kinase, particularly differences between active and inactive kinase states. Much less detail has been provided to describe how the different kinase states impacts substrate behavior. To begin to understand how key residues of the A-loop are interacting, we plot the contact map between the B-Raf (or KSR1) and MEK1 A-loops for the active wild-type B-Raf, inactive wild-type B-Raf, and KSR1 systems (Fig. 6). The contact maps for the other active pThr599/pSer602 wild-type B-Raf and B-Raf V600E systems are also provided (Fig. S17). In all systems, MEK1 Val224 makes extensive contacts with the A-loop of B-Raf and KSR. In particular, the backbone of Val224 forms a stable hydrogen bond with the backbone of inactive B-Raf Glu615 and KSR1 Leu769. In active B-Raf systems, the Glu615-Val224 hydrogen bond is transient and instead other hydrogen bonds form between Val224 and side chain atoms in B-Raf 612–614.

The contact maps also show Phe223 of MEK1 makes more contact with the A-loop of KSR1 and active B-Raf than with the A-loop of inactive B-Raf (Fig. 6a). The main chain dihedral angles for Ser222 and Phe223 of MEK1 along with the first two sidechain dihedral angles for Phe223 show that Ser222 adopts more heterogeneous backbone dihedral angles in active systems (Figs. 6b, S15) and in the KSR1 system (Fig. 6c) than the inactive systems. The main chain dihedral angles of Phe223 are constant across all systems, but the first two sidechain dihedral angles show much more variability in the active B-Raf and KSR1 systems than for the inactive wild-type B-Raf system. Sequence alignment (Fig. S18) of MEK1 and MEK2 from various organisms show that the SFV residues are largely conserved among vertebrates, suggesting that the interactions detailed above describe a general mechanism for how MEK1 docks to Raf and KSR proteins. The increased contact of Phe223 with the A-loop of B-Raf and KSR1, the increased range of dihedral angles for Ser222 and Phe223, and the larger MEK1 A-loop RMSF of the active B-Raf and KSR1 systems indicate that active B-Raf and KSR1 have a similar impact on the MEK1 A-loop, and that this impact is different than that of inactive B-Raf.

### MEK1 serine phosphorylation

**Fig. 7:**
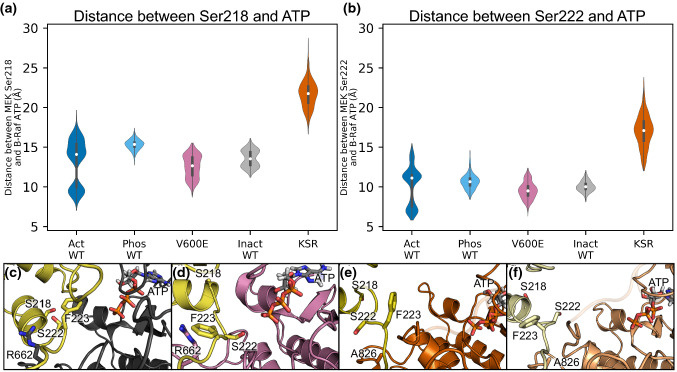
Active B-Raf/MEK1 dimer interface allows the -OH group of MEK Ser222 to be positioned for phosphorylation. Distance between ATP in B-Raf (or KSR1) and MEK1 **a** Ser218 and **b** Ser222 for all systems. **c** Representative snapshots of inactive B-Raf/MEK1 with Ser222 oriented towards MEK1 and Phe223 oriented towards B-Raf. **d** Representative snapshot of active B-Raf V600E with Ser222 oriented towards ATP and Phe223 oriented towards MEK1. **e**, **f** Ser222 and Phe223 can switch orientations in the KSR1/MEK1 system despite KSR1 not adopting an active kinase configuration

From the crystal structures, Ser222 is positioned closer to ATP in B-Raf (or KSR1) than Ser218. To delineate a catalytic phosphorylation reaction in the ATP-binding pocket of B-Raf (or KSR1), we calculated the distance from the C_α_ of these residues to the γ-phosphate of the ATP in B-Raf (or KSR1) for Ser218 and Ser222 (Fig. 7a, b). A time series of the distances show significant fluctuations in the distance during the simulations (Fig. S19). Our simulations confirm that Ser222 is usually closer to ATP, indicating that this residue is likely the first serine to be phosphorylated in the activation of MEK1. For the inactive wild-type B-Raf/MEK1 system, a representative snapshot of the position of Ser218 and Ser222 relative to ATP shows that Ser222 is closer to ATP than Ser218, but that the sidechain -OH group is oriented towards MEK1, not in the proper orientation to be phosphorylated (Fig. 7c). Instead, Phe223 is oriented towards the B-Raf/MEK1 dimer interface. Representative snapshots for all configuration subfamilies show that this orientation of Ser222 and Phe223 is conserved for all inactive B-Raf systems (Fig. S20). For the active B-Raf V600E/MEK1 system, the orientation of Ser222 and Phe223 has swapped when Ser222 is closest to ATP (Fig. 7d). Ser222 is positioned with its oxygen atom of the sidechain -OH group within 5 Å of the γ-phosphate of ATP, while Phe223 has rotated to orient towards MEK1. Representative snapshots for the active B-Raf systems show that Ser222 and Phe223 can adopt a wider range of orientations when B-Raf is active compared to inactive (Fig. S20). Finally, for the KSR1/MEK1 system, the representative snapshots show that orientations of Ser222 and Phe223 can switch, despite KSR1 not being activated (Fig. 7e, f).

In Fig. 7c, d, Arg662 of B-Raf is located within the “A-loop pocket” of MEK1, positioning it near the ATP molecule in MEK1. This implies that the phosphorylation of Ser222 does not require the movement of Arg662 to the outside of the “inhibitory helix” of MEK1 (shown in Fig. 5e). This indicates that the movement of Arg662 is a result, not a cause, of increased RMSF values of the A-loop of MEK1 as seen in Fig. S13. Instead, we propose that the different positions of the MEK1 P-rich loop between the active and inactive B-Raf/MEK1 systems, shown in “Structural changes of MEK1” section, is the source of the increased RMSF in active B-Raf/MEK1. In the KSR1/MEK1 systems, the P-rich loop can adopt a similar orientation as is seen in the inactive B-Raf/MEK1 systems; therefore, we propose that the shorter Ala826 residue (compared to Arg662 in B-Raf) is the cause of the increased RMSF values of the MEK1 A-loop.

**Fig. 8:**
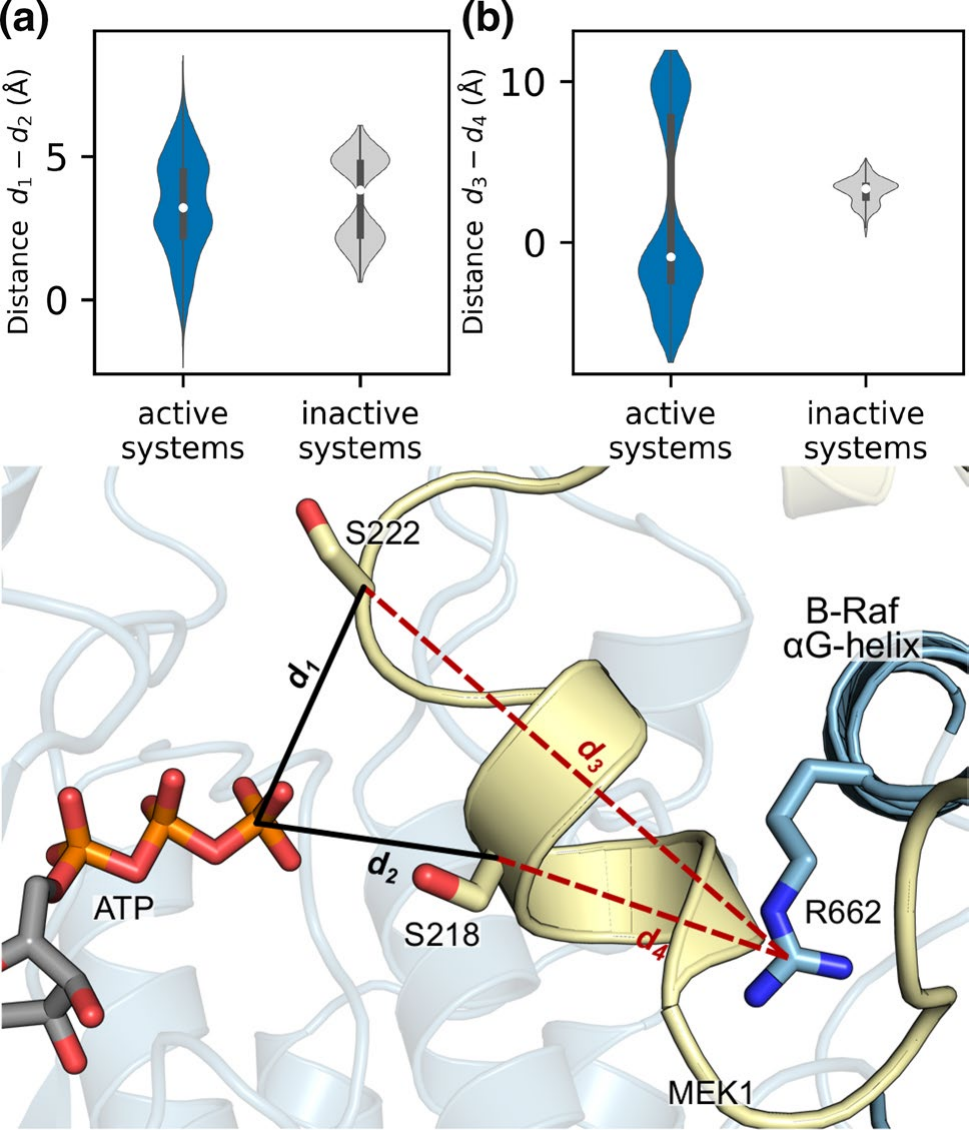
Ser218 can move closer to ATP when B-Raf Arg662 moves to the outside of the N-terminal helix of the A-loop in MEK1. **a** Distance between Ser222 and ATP (*d*_*1*_) minus the distance between Ser218 and ATP (*d*_*2*_). Positive values indicate that Ser222 is closer to ATP than Ser218. **b** Distance between Arg662 and Ser222 (*d*_*3*_) minus the distance between Arg662 and Ser218 (*d*_*4*_). Positive values indicate Arg662 is inside the A-loop of MEK1. Bottom: diagram indicating the distances measured in (**a**, **b**)

There are events when the distances between Ser218/Ser222 and the ATP molecule in B-Raf are approximately equal or Ser218 can be closer than Ser222, as can be seen in the “Act WT 1” and “Act WT 3” plots of Fig. S19. We explored these instances further and found that Ser218 was closer to ATP than Ser222 for approximately 5% of all active systems, but this never occurred in inactive systems, Fig. 8a. Furthermore, Ser218 was closer to ATP than Ser222 only when Arg662 was positioned on the outside of the MEK1 A-loop “inhibitory helix.” We used the distance between C_ζ_ of Arg662 and C_α_ of Ser218/Ser222 to determine the position of Arg662 and found that it was outside of the MEK1 A-loop for 60% of the active systems but was never outside the A-loop for inactive systems, as can be seen in Fig. 8b. This further indicates that Ser222 is more likely to be phosphorylated first in the activation of MEK1 but does not preclude the possibility a small subset of the population in which Ser218 is phosphorylated first. Regardless of whether Ser218 is phosphorylated first or second, the movement of Arg662 to the outside of “inhibitory helix” appears to be an important step in repositioning the MEK1 A-loop to achieve full MEK1 activation.

## Discussion

To help understand MEK1 activation, we have provided a detailed description of the protein dynamics, structure, and interface for active B-Raf/MEK1, inactive B-Raf/MEK1, and KSR1/MEK1 dimers. Our analysis highlighted structural differences in the position of the MEK1 P-rich loop and the importance of the αG-helix not only in contributing to dimerization but also for its impact on the MEK1 A-loop. These results could help guide the development of MEK inhibitors.

**Fig. 9:**
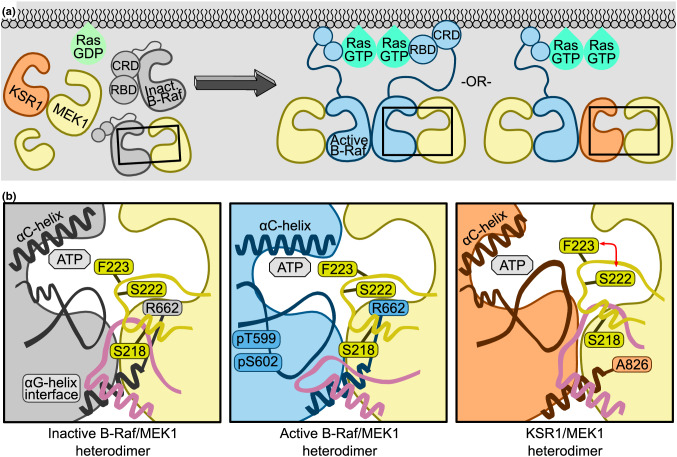
Features of B-Raf/MEK1 and KSR1/MEK1 dimer interfaces that play key role in MEK activation scenarios by B-Raf (shown in Fig. 10) and their comparison with KSR1. (**a**, Left) Ras is inactive, as is B-Raf (in gray color), MEK1 (yellow) and KSR1 (orange). The monomers are in equilibrium with B-Raf/MEK1 and KSR1/MEK1 dimers. (Right) Ras is active; B-Raf and KSR1 are recruited to the membrane, B-Raf is activated through side-to-side dimerization with either B-Raf or KSR1. Active B-Raf (blue), KSR1 and MEK1 can then form quaternary complexes. **b** Close up views of the dimer interfaces outlined in black boxes in (**a**). (Left) Inactive B-Raf is unable to phosphorylate MEK1 due to the collapsed MEK1 A-loop (yellow loop) stabilized by interactions with B-Raf A-loop (black loop), MEK1 P-rich loop (pink loop) residues, and with B-Raf Arg662. (Middle) The B-Raf A-loop extends and the MEK1 P-rich loop relocates, allowing increased MEK1 A-loop flexibility. (Left) In the KSR1/MEK1 dimer, the MEK1 P-rich loop interacts with both KSR1 and MEK1 A-loops, as in inactive B-Raf/MEK1. Flexibility of the MEK1 A-loop is due to the small Ala826 in KSR1 replacing the large Arg826 in B-Raf, allowing MEK1 Phe223 and Ser222 to switch positions

Raf family proteins have long been recognized as one of the primary catalysts for MEK phosphorylation. Our simulations highlight differences between the active B-Raf/MEK1 and inactive B-Raf/MEK1 and suggest a mechanism whereby B-Raf activation leads to phosphorylation of MEK1. Inactive B-Raf/MEK1 dimers are in equilibrium with KSR1/MEK1 dimers as well as monomeric B-Raf, MEK1, and KSR1 (Fig. 9a). The inactive B-Raf/MEK1 heterodimer is stabilized by extensive C-lobe contacts (Fig. 9b, left panel). These include the αG-helix and A-loop interactions, and MEK1 P-rich loop to B-Raf A-loop. In this inactive B-Raf/MEK1 heterodimer, both Ser218 and Ser222 are oriented away from the B-Raf’s ATP, and the Phe223 sidechain is at the dimer interface. In addition, the MEK1 P-rich loop makes extensive contacts with both inactive B-Raf and MEK1 A-loops. In response to external stimuli such as epidermal growth factor, Ras-GDP is converted to Ras-GTP. Ras-GTP can dimerize (or form nanoclusters) and recruit B-Raf monomers or B-Raf/MEK1 dimers to the membrane through interactions between Ras and the RBD of B-Raf. This relieves B-Raf autoinhibition and allows for the formation of side-to-side B-Raf/B-Raf and B-Raf/KSR1 dimers. The dimers stabilize the active B-Raf, characterized by an inward motion of the αC-helix and autophosphorylation of Thr599 and Ser602. It is likely that B-Raf autophosphorylation requires dissociation of B-Raf and MEK1 to allow ADP/ATP exchange and extension of the B-Raf A-loop. In fully activated B-Raf, the substrate MEK1 can dock to active B-Raf, promoting either a MEK1/B-Raf/B-Raf/MEK1 or MEK1/B-Raf/KSR1/MEK1 quaternary complex (Fig. 9a) [20]. The resulting active B-Raf/MEK1 interface exhibits different binding between the A-loop of B-Raf, the A-loop of MEK1 and the P-rich loop of MEK1 as compared to the inactive B-Raf/MEK1 interface. The extended A-loop of B-Raf makes fewer contacts with residues in the MEK1 P-rich loop. The P-rich loop can move from its location along the side of MEK1 C-lobe (near MEK1 αD-helix and A-loop) to a location at the bottom of the C-lobe (near MEK1 αH and αG-helices) (Fig. 9b, middle panel).

**Fig. 10:**
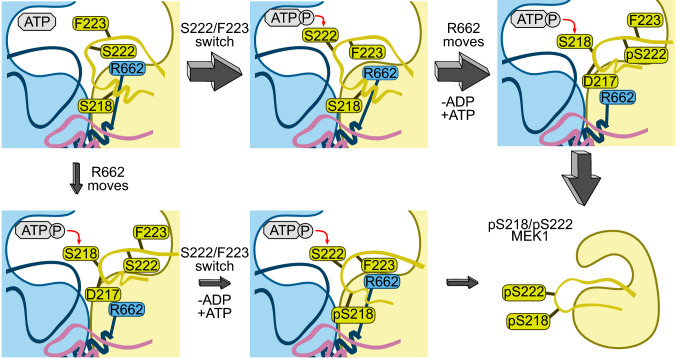
Proposed mechanism for the activation of MEK1 by B-Raf. B-Raf can activate MEK1 through two routes. The primary route involves phosphorylation of Ser222 first, followed by phosphorylation of Ser218. The secondary route reverses the order of phosphorylation. Route preference, as to whether S222/F223 flip their orientation first, or R662 moves, reflects the relative populations of the conformational states, which depends on the relative stabilities. We suggest that route shown on top is more highly populated

Crystal structures show that Ser222 is located closer to the ATP-binding pocket of B-Raf suggesting that phosphorylation of Ser222 is more likely to occur first. Recent experiments on kinase-dead MEK1 in the presence of active B-Raf indicate that both singularly phosphorylated pSer218 MEK1 and pSer222 MEK1 can occur, and that MEK1 pSer222 was more common than p218 MEK1. In addition, MEK inhibitors differentially impact single-site MEK phosphorylation [61]. These results suggest that either Ser218 or Ser222 can be phosphorylated first, with Ser222 being more likely in most MEK1 activation events. Our simulations offer additional evidence to support this conclusion and suggest two possible pathways by which MEK1 activation can occur. Both pathways begin with the active B-Raf/MEK1 dimer as depicted in the middle panel of Fig. 9b. In the first pathway (Fig. 10, top path), relocation of the MEK1 P-rich loop disrupts interactions between it and the MEK1 A-loop, allowing increased fluctuations of the A-loop. This increases the population of conformations in which the sidechains of Phe223 and Ser222 flip positions, orienting Ser222 for phosphorylation. Ser222 phosphorylation and ATP regeneration facilitate the A-loop of MEK1 reorientation to position Ser218 for phosphorylation. A-loop reorientation may be assisted by the B-Raf Arg662 shift to the outside of the inhibitory helix or dissociation and re-binding of MEK1 pSer222. The movement of B-Raf Arg662 is stabilized by the formation of salt bridges between B-Raf Arg662 and Asp663 with MEK1 Asp217 and Arg189, respectively. Once both Ser222 and Ser218 are phosphorylated, MEK1 is primed for activation of ERK. We propose that this mechanism is the primary means by which MEK1 is phosphorylated. The simulations also suggest a second pathway (Fig. 10, bottom path) in which Arg662 of B-Raf is on the outside of the inhibitory helix, positioning Ser218 to be phosphorylated first. In the phosphorylated Ser218 and ATP regeneration state, the MEK1 A-loop can be repositioned, with Phe223 and Ser222 again flipping positions to orient Ser222 towards ATP. These proposed mechanisms and simulation results accurately reflect and extend current knowledge of MEK1 activation. Our proposed mechanism could also serve as a basis for exploring why MEK inhibitors have different impacts on the phosphorylation of Ser218 and Ser222.

Our results also provide additional details into the role of KSR in MEK activation. KSR has been described as an active kinase [47, 57], a scaffold [48] or a transactivator [45]. *The results of our simulations indicate that if KSR1 is to function as an active kinase, it would be a low probability event*. The lack of N-lobe contacts between MEK1 and KSR1 in the crystal structures is consistent with our simulations. Significant rearrangement of the dimer interface would be necessary for the KSR1-ATP pocket being in proximity of Ser218 and Ser222 of MEK1. This could be achieved through allosteric effects due to KSR/Raf interaction or additional scaffolding proteins such as 14-3-3. When the N-lobes of MEK1 and KSR1 are closer, our results suggest that the innate flexibility of the MEK1 A-loop in the KSR1/MEK1 heterodimer would allow proper serine orientation to achieve phosphorylation (Fig. 9b, right panel). The innate flexibility of the A-loop derives from the presence of short alanine residues at the N-terminal end of KSR1 αG-helix, which do not form strong interactions with MEK1 A-loop, unlike in the B-Raf/MEK1 system. In addition, if the A-loop of KSR1 were to adopt the “extended” configuration of other active kinases, our results predict that the P-rich loop of MEK1 would move in concert with the A-loop of KSR1. This would pull the P-rich loop away from the A-loop of MEK1 and further increase its flexibility. Our simulations also provide information that could both support and oppose the model of KSR serving as a scaffolding protein for MEK phosphorylation. In this model, Raf from an active Raf dimer phosphorylates the MEK of a Raf/KSR/MEK ternary “scaffold unit.” For this model to be accurate, it requires significant space between the KSR/MEK dimer interface for the active Raf to approach. Our simulations show that the N-lobes of MEK1 and KSR1 move apart and that the MEK1 A-loop is flexible. Both results indicate that the KSR1/MEK1 interface provides higher access probability to the MEK1 A-loop than the B-Raf/MEK1 interface; however, our results do not indicate any large-scale reorientation of the MEK1 A-loop that would be necessary for B-Raf to approach. In the KSR1/MEK1 system, Ser222 is buried due to the position of KSR1 A-loop. Ser218 is closer to the surface of MEK1; however, it is located underneath the outward-oriented αC-helix of MEK1. Due to these steric constraints, it would be difficult for a second B-Raf A-loop to approach the KSR1/MEK1 dimer interface. The model of KSR as a transactivator of Raf, wherein KSR/MEK heterodimers allosterically activate Raf leading to the phosphorylation of a substrate MEK by Raf, is a more recent explanation of KSR’s role in the MAPK pathway. Our results do not directly address this model but could serve as a basis for future exploration. Simulations of KSR1 monomers, KSR1/B-Raf heterodimers, and MEK1/KSR1/B-Raf ternary complexes, in addition to results from this work, could be used to address this model more directly.

Early studies of the function of MEK1 P-rich loop present apparently conflicting results regarding the necessity of this loop for the formation of Raf-1/MEK1 heterodimers. Catling et al. in 1995 found that deletion of MEK1 residues 270–307 (MEK1Δ270-307) abrogated Raf-1/MEK1Δ270-307 binding in CCL39 cells transiently transfected with MEK1 from Rat1 cells (residue 307 in rat cells corresponds to the final proline in the P-rich loop, in human MEK1 the final proline is also residue 307) [62]. Dang et al. in 1998 found that deletion of residues 265–301 of human MEK1 did not abrogate Raf-1/MEK1Δ265-301 binding. Since rat and human MEK1 proteins contain nearly identical sequences in the P-rich loop, comparison between the two different systems is reasonable [63]. The discrepancy between these two results (no Raf-1 interaction with MEK1Δ270-307, but with MEK1Δ265-301) can be explained by our simulations. The contact maps between MEK1 P-rich loop and B-Raf A-loop (Fig. 4) show that MEK1 residues 305–307 make multiple contacts with the A-loop of B-Raf. Based on our contact maps and the differences between the two deletion segments used in the two experiments, we would expect an interaction between Raf-1 and MEK1Δ265-301 (which maintains the key 305–307 residues) and no interaction between Raf-1 and MEK1Δ270-307 (which does not include these residues). The loop’s flexibility and position on the surface of the protein make crystallization with these residues difficult, but mutations or short deletions could be used to verify the importance of the P-rich loop/B-Raf A-loop interactions for dimerization. Particularly, mutation of MEK1 Arg305 and B-Raf H608G or deletion of MEK1 residues 301–307 (MEK1Δ301-307) or B-Raf residues 605–611 (B-RafΔ605-611) could be used to change the B-Raf/MEK1 interface and abrogate interaction.

Our results highlight the importance of the αG-helix region to the structure and function of the B-Raf/MEK1 and KSR1/MEK1 heterodimers. The residues at the N-terminal end of the αG-helix of B-Raf are Asn661 and Arg662 (“large”), while for KSR1 the residues are Ala825 and Ala826 (“small”). This “large” vs. “small” difference (which is highly conserved across species) has recently been highlighted to describe how the MEK inhibitor (MEKi) trametinib favors binding of KSR1/MEK1 over B-Raf/MEK1 [10]. Trametinib binds to the allosteric pocket in MEK1 and, unlike other MEKi, extends into a pocket at the KSR1/MEK1 interface, directly interacting with KSR1 Ala825. The longer B-Raf Arg662 residue would cause steric clashes with trametinib at the B-Raf/MEK1 interface. Reciprocal mutations of these residues allowed B-Raf to mimic the behavior of KSR1 with respect to MEK1 interaction in the presence of trametinib and vice versa. Our results reinforce the importance of these residues due to their impact on the flexibility of the MEK1 A-loop and suggest that a MEKi inhibitor that interacts with Arg662 could improve B-Raf/MEK1 interaction and prevent MEK1 activation. This proposed inhibitor could prevent Arg662 from relocating away from the MEK inhibitory helix, preventing the phosphorylation of Ser218 and ‘trapping’ inactive B-Raf/MEK1 complex.

Our results are based on microsecond long simulation trajectories, while activation-related kinase conformational changes happen on the millisecond timescale [64, 65] and phosphorylation via kinases can take milliseconds to seconds [66]. Therefore, care must be taken in interpreting results from MD simulations. Our proposed mechanism for B-Raf phosphorylation of MEK1 aligns well with previous descriptions of MEK1 activation based on in vitro and in vivo experiments [21, 24, 37, 61]. Our results support a description of how local changes in residue backbone and sidechain orientation can result in B-Raf and MEK1 adopting conformations that are favorable to the initiation of the phosphorylation reaction. Our observed local changes in MEK1 and KSR1 can be compared to those in the B-Raf/MEK1 simulations, and inferences can be drawn based on the similarities and differences in the KSR1/MEK1 and B-Raf/MEK1 dimer interfaces. We cannot, however, draw definitive conclusions about the KSR1/MEK1 dimer due to the smaller set of experiments with which to compare our results (as opposed to the wealth of information about B-Raf/MEK1 dimers) and the smaller set of crystal structures available for KSR1 (that could impact our starting configurations and trap the protein in local energy minimum).

## Conclusions

We have provided a detailed comparison of the interface between MEK1 and active B-Raf, inactive B-Raf, and KSR1. Our results point to (i) an important and not previously described role for the MEK1 P-rich loop as both a key source of interactions for complex formation and to regulate the flexibility and thus phosphorylation of the MEK1 A-loop. We also suggested (ii) how KSR1 could act as both an active kinase and a scaffolding protein. The KSR1/MEK1 interface results in a much more flexible MEK1 A-loop as compared to that of B-Raf/MEK1. This increased flexibility would not be sufficient by itself to allow for either direct activation of MEK1 by KSR1 or for active B-Raf to approach a KSR1/MEK1 “scaffold” dimeric unit. However, low population events (i.e., increased flexibility causing the N-terminal A-loop helix to unfold) could lead to either of these scenarios.

Overall, our results suggest that (iii) even though direct phosphorylation of MEK1 by KSR1 is possible, it is unlikely compared to phosphorylation by B-Raf. Instead, our results suggest that the primary role of the KSR1/MEK1 heterodimer is either a scaffold or an allosteric activator, and they offer how, providing a mechanism. As a scaffold, KSR1 from a KSR1/MEK1 heterodimer interacts with B-Raf through a side-to-side interface. MEK1 from this Raf/KSR1/MEK1 ternary scaffolding unit is then translocated to an active B-Raf dimer and phosphorylated by B-Raf from the dimer [48, 57]. MEK1 can relieve allosterically KSR1 autoinhibition, permitting KSR1 formation of a side-to-side heterodimer with a B-Raf monomer recruited to the membrane by Ras. The stabilized active configuration of B-Raf can then phosphorylate a second MEK1 kinase [45]. The structural and functional mechanism that we decipher clarifies MEK1 activation scenarios and can help guide drug discovery for MEK1, a vital component of the MAPK pathway.

MAPK encompasses many additional proteins collaborating in complex formation and stabilization [1]. Further, each of the three kinase domains discussed here, resides on proteins that contain additional structured and disordered regions that are integral to proper cell function. For instance, recent simulations have revealed that a basic motif in the disordered region N-terminal of the B-Raf kinase domain is implicated in stabilizing the B-Raf/B-Raf homodimer [17]. As computational power grows and new tools become available to predict protein structure [67, 68], new insights into the impact of larger assemblies will be possible [1].

## Materials and methods

### Modeling of B-Raf/MEK1 and KSR1/MEK1 heterodimers

Initial coordinates for the B-Raf and MEK1 kinase domain dimers were adopted from crystal structures (PDB IDs: 4MNE, 6U2G, 6PP9). The B-Raf kinase domain (residues 449–720) exhibited either an active (4MNE) or inactive (6U2G, 6PP9) configuration, while the MEK1 kinase domain (residues 40–317 or residues 63–317) was in an inactive configuration. The best resolved B-Raf /MEK1 complex was extracted from those crystal structures that contain B-Raf/MEK1 quaternary complexes. Except for residue 600, any mutated residues present in the crystal structures were changed back to the wild-type sequence. Residue 600 was either kept as the wild-type Val or mutated to Glu to create the oncogenic B-Raf V600E. In addition, select simulations contained phosphorylated B-Raf A-loop with pThr599/pSer602. Initial coordinates for the KSR1/MEK1 complex were adopted from crystal structure (PDB ID: 7JUW). Loop segments that were missing from the crystal structures were constructed with SWISS-MODEL [69], using available loop conformations in a database from PDB as a template. A summary of the PDB entries used to create the initial configurations is provided in Table S1.

ATP analogs in B-Raf, KSR1, and MEK1 were replaced with ATP and Mg^2+^ and MEK1 inhibitors were removed from the structure. B-Raf structures that did not contain a ligand in the binding pocket had ATP/Mg^2+^ added using the binding between B-Raf and AMP-PCP (an analog of ATP) in 6U2G as a template. This created a system containing the B-Raf/MEK1 (or KSR1/MEK1) heterodimer with ATP and Mg^2+^ present in each protein. Details of each starting configuration are summarized in Table S2.

### MD simulation protocol

All-atom MD simulations were conducted using the NAMD package [70] with the updated CHARMM force field [71, 72]. Our simulations closely followed the same protocol as in our previous works [15, 17, 35, 59, 73–89]. The explicit TIP3 water model was used to solvate a periodic box of $\sim 120\times 120\times 120 {\AA }^{3}$. Water molecules within 2.6 Å of the proteins were removed to prevent atom collapse. Salt ions (sodium and chlorine) were added to generate a final ionic strength of $\sim 100 \mathrm{mM}$ and neutralize the system. A series of minimization cycles were performed for the solvents including ions with a harmonically restrained protein backbone until the solvent reached 310 K. Next, dynamic cycles were performed while gradually releasing the harmonic constraints on the protein backbone. The long-range electrostatics calculation was performed using the particle mesh Ewald (PME) method. During productions runs, a Langevin thermostat maintained a constant at 310 K temperature and a Nosé–Hoover Langevin piston pressure control sustained the pressure at 1.01325 bar (1 atm) with the NPT condition. A 2 fs time step was used for $5\times {10}^{8}$ steps for all simulations. A total of 10 µs simulation were performed for 10 systems, each with 1 µs simulation time. Trajectory information was collected every $5\times {10}^{4}$ steps (100 ps).

### Analysis methods

Statistics presented in “Results” section were calculated based on the final 500 ns of the trajectories, unless otherwise indicated (i.e., Fig. S8 which shows how protein–protein contact area evolves over time). Principal component analysis (PCA) was performed using Bio3D [90]. The distance between atoms and dihedral angles were calculated using MDTraj [91]. Root mean square fluctuations (RMSFs) were calculated in CHARMM [92]. Protein–protein contact area was calculated using solvent accessible surface area techniques provided in the dr-sasa analysis package and PDBePISA [93, 94]. Protein images were created using Pymol [58]. Representative models from the ensemble trajectory results were selected using the Ensemble Cluster [95] implementation available in Chimera [96]. Protein sequence alignment was accomplished using Clustal Omega [97].

## Supplementary Information

Below is the link to the electronic supplementary material.

Supplementary file1 (DOCX 8811 KB)

Supplementary file2 (MP4 18495 KB)

Supplementary file3 (MP4 14531 KB)

Supplementary file4 (MP4 13610 KB)

Supplementary file5 (MP4 16106 KB)

Supplementary file6 (MP4 13442 KB)

Supplementary file7 (MP4 12818 KB)

Supplementary file8 (MP4 19086 KB)

Supplementary file9 (MP4 14001 KB)

Supplementary file10 (MP4 13298 KB)

## Data Availability

The calculations had been performed using the high-performance computational facilities of the Biowulf PC/Linux cluster at the National Institutes of Health, Bethesda, MD (https://hpc.nih.gov/). Derived data supporting the findings of this study are available from the corresponding author on request.
