# Supplementary material for: The mechanism of activation of MEK1 by B-Raf and KSR1
Source: Cell Mol Life Sci. 2022 May 4;79(5):281. doi: 10.1007/s00018-022-04296-0 (PMC9068654; doi:10.1007/s00018-022-04296-0)
Supplement: Supplementary file 1 — Supplementary file1 (DOCX 8811 KB) [file 18_2022_4296_MOESM1_ESM.docx]

**Supplementary Information**

## Ryan C. Maloney^1^ • Mingzhen Zhang^2^ • Yonglan Liu^1^ • Hyunbum Jang^2^ • Ruth Nussinov^2,3,^*

^1^ Cancer Innovation Laboratory, National Cancer Institute, Frederick, MD 21702, USA

^2^ Computational Structural Biology Section, Frederick National Laboratory for Cancer Research, Frederick, MD 21702, USA

^3^ Department of Human Molecular Genetics and Biochemistry, Sackler School of Medicine, Tel Aviv University, Tel Aviv 69978, Israel

* Author for correspondence: R.N.

Tel: 1-301-846-5579

E-mail: [NussinoR@mail.nih.gov](mailto:NussinoR@mail.nih.gov)

**Table S1** Summary of PDB entries used to create the initial configurations for simulations.

| PDB ID | System | B-Raf chain | Mutated residues | | Missing residues | | MEK chain | | Mutated residues | | Missing residues |
| --- | --- | --- | --- | --- | --- | --- | --- | --- | --- | --- | --- |
| 4MNE | B-Raf:MEK1 | B | none | | 465-468 | | B | | none | | 275-305 |
| 6U2G | B-Raf:MEK with AMP-PCP bound to B-Raf | B | none | | 449-450  603-610 | | A | | none | | 275-306 |
| 6PP9 | B-Raf:MEK1 | A | none | | none | | B | | Ser218Ala  Ser222Ala | | 275-306 |
| PDB ID | System | KSR chain | Mutated residues | | Missing residues | | MEK chain | | Mutated residues | | Missing residues |
| 7JUW | KSR1:MEK1 in complex with AMP-PNP | B | none | | 761-764 | | C | | none | | 275-306 |
|  |  | | |  | |  | |  | |  | |

**Table S2** Initial configurations of the simulated systems of dimeric B-Raf/MEK1 or KSR1/MEK1.

|  |  | B-Raf or KSR1 | | MEK1 |
| --- | --- | --- | --- | --- |
| Dimer | PDB  ID | αC-helix | A-loop | N-terminal helix |
| MEK1 with active B-Raf wild-type: | 4MNE | in | extended | no |
|  | 4MNE | in | extended | no |
|  | 4MNE* | in | extended | no |
|  |  |  |  |  |
| MEK1 with active pT599/pS602 BRAF: | 4MNE | in | extended | no |
|  |  |  |  |  |
| MEK with B-Raf V600E: | 4MNE | in | extended | no |
|  | 4MNE* | in | extended | no |
|  |  |  |  |  |
| MEK1 with inactive B-Raf wild-type: | 6U2G | out | collapsed | no |
|  | 6PP9 | out | collapsed | yes |
|  |  |  |  |  |
| MEK1 with KSR1 | 7JUW | out | collapsed | no |
|  | 7JUW | out | collapsed | yes |
| * The initial configuration for B-Raf in these simulations were obtained by extracting B-Raf from the PDB entry and performing 1 µs simulations on monomeric B-Raf. The final structure from the simulation was then docked back on to MEK from the PDB entry, using the original B-Raf coordinates as a template for fitting the new structure. | | | | |


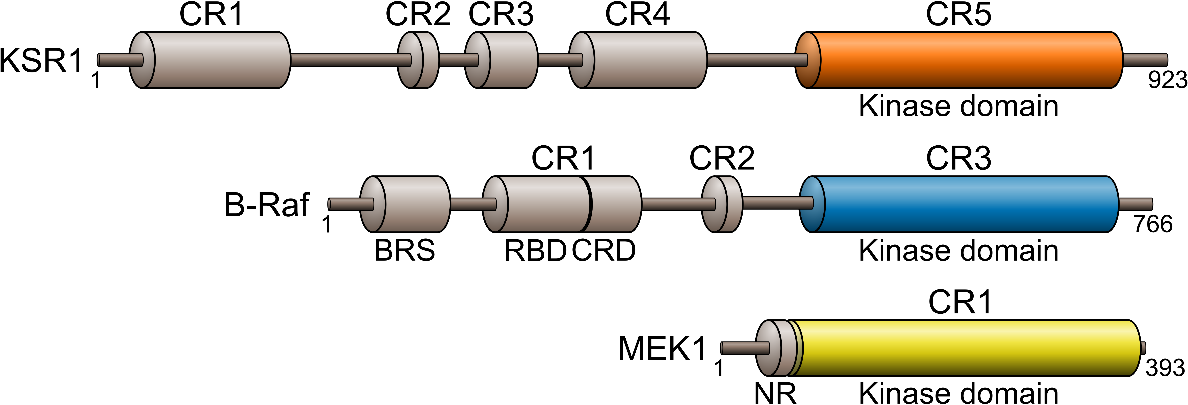


**Fig. S1** Conserved regions (CR) of KSR1, B-Raf, and MEK1. BRS is a B-Raf specific binding region, RBD is the Ras-binding domain, CRD is the cysteine-rich domain, and NR is the MEK1 N-terminal regulatory helix.

**Fig. S2** Sequence alignment of MEK1, KSR1, and B-Raf. Highlighted sequences denote key secondary structural features (β-strands in yellow, α-helix in pink) and the location of the activation loop (green). Residues that undergo phosphorylation in the activation of the kinase are shown in red.

β1 β2 β3 αC

MEK1 63 -------LKDDDFEKISELGAGNGGVVFKVSHKPSGLVMARKLIHLEIKPAIRNQIIREL 115

KSR1 601 VYLQEWDIPFEQVELGEPIGQGRWGRVHRGRWH--GEVAIRLLEMDGHNQDHLKLFKKEV 658

B-Raf 449 ----DWEIPDGQITVGQRIGSGSFGTVYKGKWH--GDVAVKMLNVTAPTPQQLQAFKNEV 502

β4 β5 αD αE

MEK1 116 QVLHECNSPYIVGFYGAFYSDGEISICMEHMDGGSLDQVLKKAGR-IPEQILGKVSIAVI 174

KSR1 659 MNYRQTRHENVVLFMGACMNPPHLAIITSFCKGRTLHSFVRDPKTSLDINKTRQIAQEII 718

B-Raf 503 GVLRKTRHVNILLFMGYST-KPQLAIVTQWCEGSSLYHHLHIIETKFEMIKLIDIARQTA 561

β6 β7 β8 activation loop

MEK1 175 KGLTYLREKHKIMHRDVKPSNILVNSRGEIKLCDFGVSGQLID**S**-------MAN**S**FVGTR 227

KSR1 719 KGMGYLHA-KGIVHKDLKSKNVFYDNGK-VVITDFGLFGISGVVREGRRENQLKLSHDWL 776

B-Raf 562 QGMDYLHA-KSIIHRDLKSNNIFLHEDLTVKIGDFGLA**T**VK**S**RW---SGSHQFEQLSGSI 617

αF

MEK1 228 SYMSPERLQ---------GTHYSVQSDIWSMGLSLVEMAVGRYPIPPPDAKELELMFGCQ 278

KSR1 777 CYLAPEIVREMTPGKDEDQLPFSKAADVYAFGTVWYELQARDW----------------- 819

B-Raf 618 LWMAPEVIRM------QDKNPYSFQSDVYAFGIVLYELMTGQL----------------- 654

αG αH

MEK1 279 VEGDAAETPPRPRTPGRPLSSYGMDSRPPMAIFELLDYIVNEPPP-KLP--SGVFSLEFQ 335

KSR1 820 ------------------------PLKNQ-AAEAS-IWQIGSGEGMKRVLTSVSLGKEVS 853

B-Raf 655 ------------------------PYSNINNRDQIIFMVGRGYLSPDLSKVRSNCPKAMK 690

αI

MEK1 336 DFVNKCLIKNPAERADLKQLMVHAFIKRSDAEEVDFAGWLCSTI 379

KSR1 854 EILSACWAFDLQERPSFSLLMDMLEKLP---------------- 881

B-Raf 691 RLMAECLKKKRDERPLFPQILASIELLARS-------------- 720


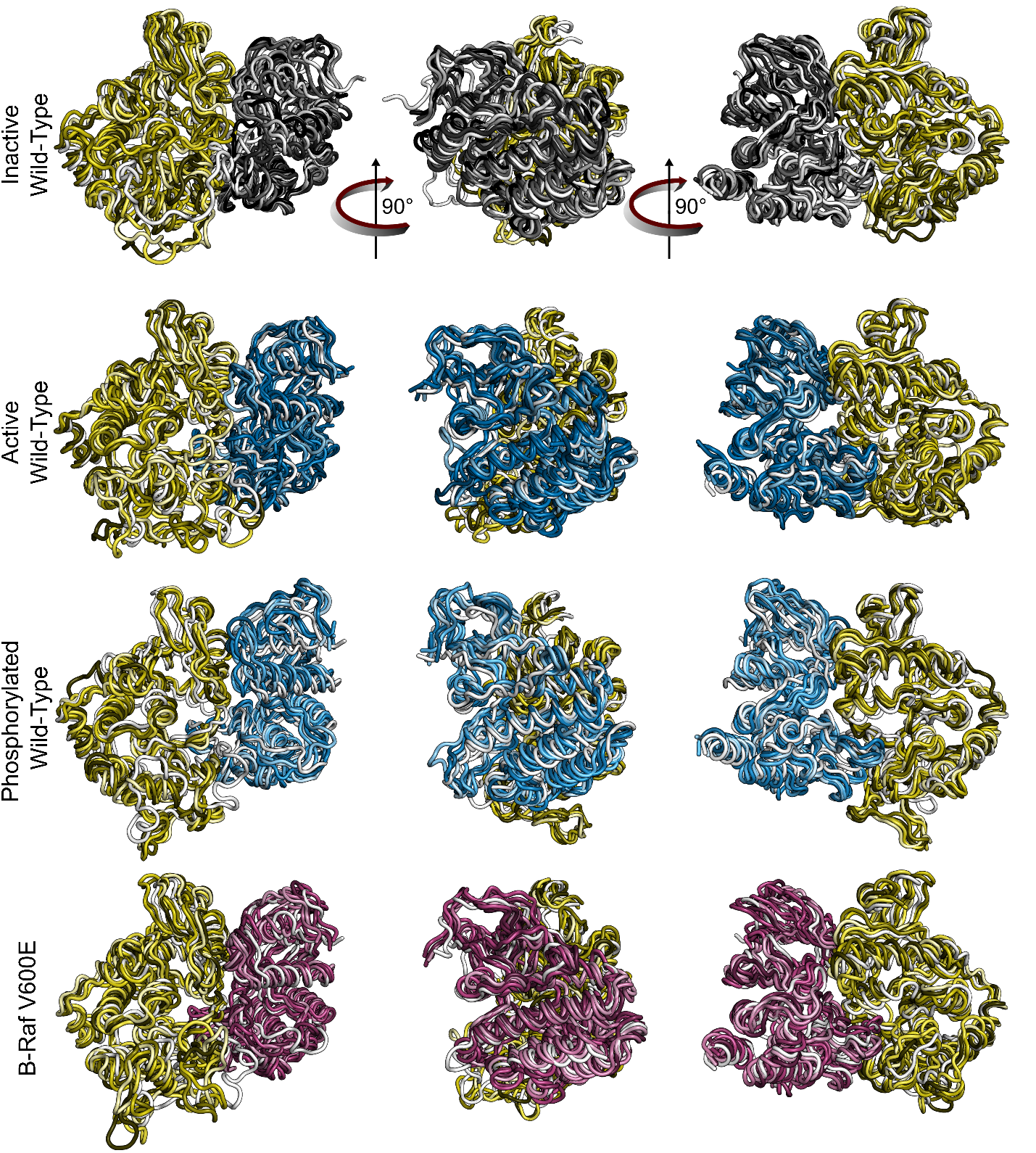


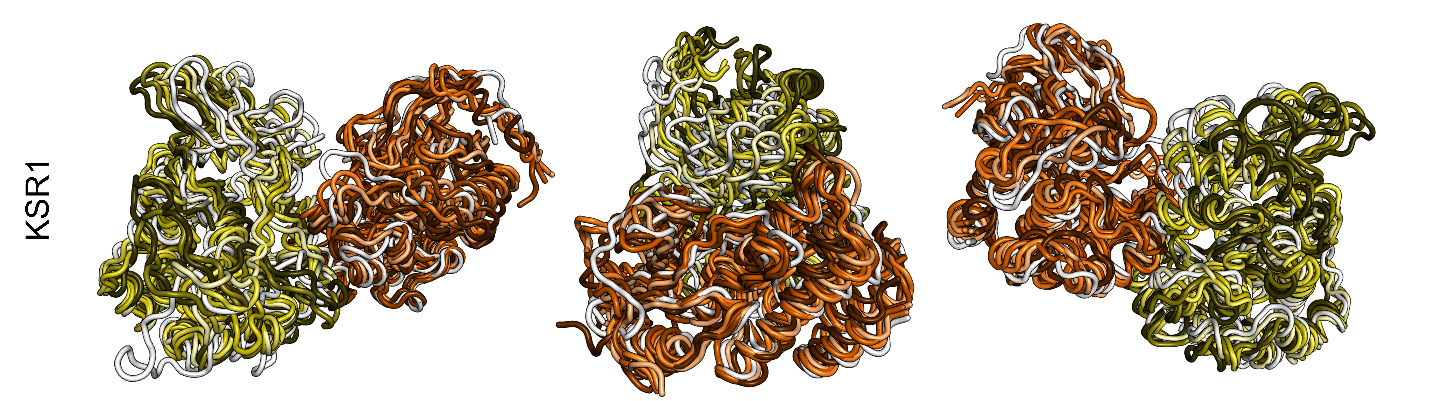
**Fig. S3** Representative protein configurations during the simulation (colored) compared to the initial configuration (white) shows that the N-lobes of B-Raf and MEK1 move closer together, while the N-lobes of KSR1 and MEK1 move apart. Colored cartoons depict representative structure of the five largest conformationally-related subfamilies from the trajectory ensemble. The darkest shade of color in each set corresponds to the most populated subfamily and each step down in color shade corresponds to the next most populated subfamily. The initial configuration is shown in white.


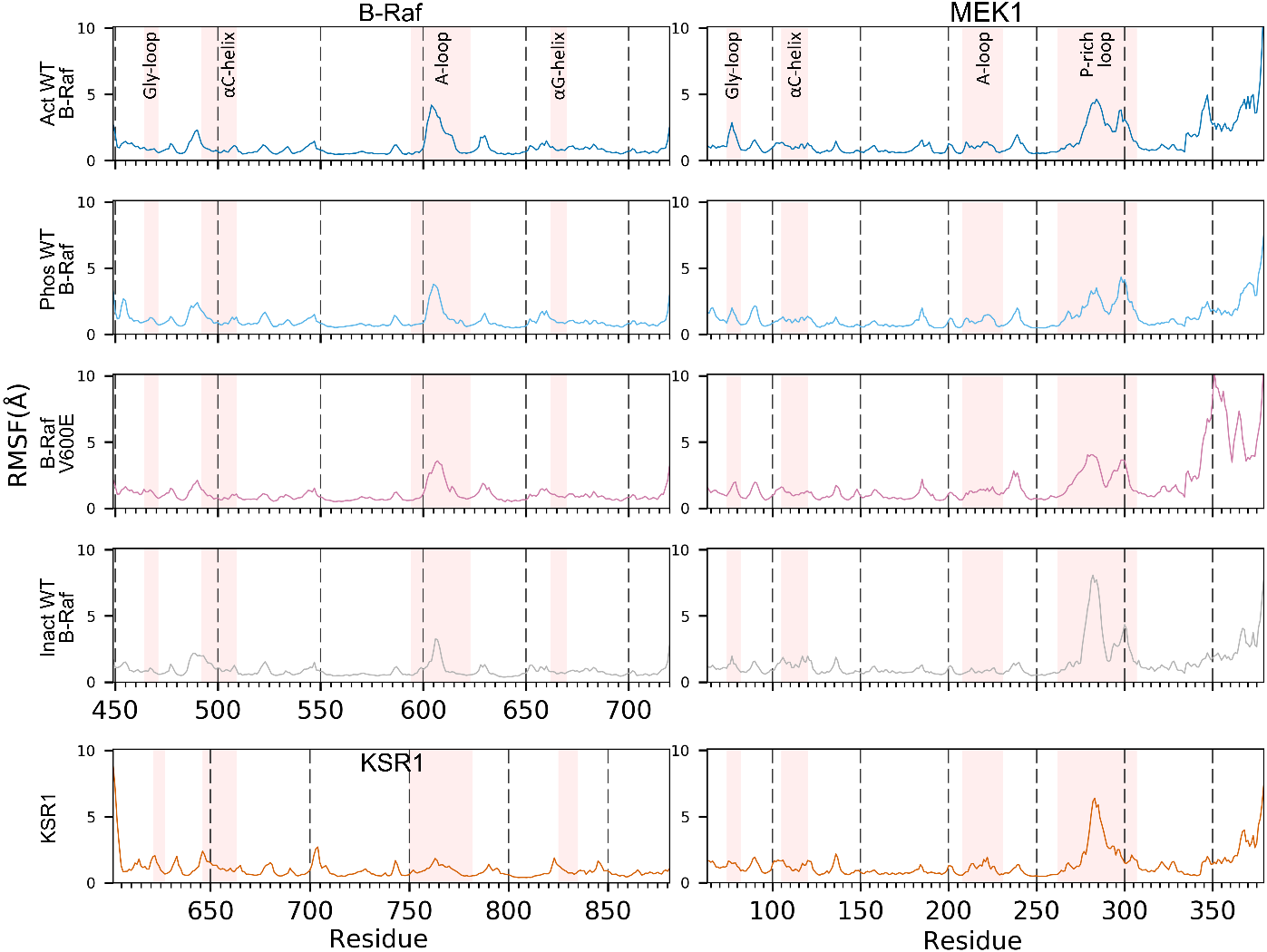


**Fig. S4** Root mean square fluctuation (RMSF) of B-Raf, KSR1, and MEK1 residues for each simulated system show that the largest residue fluctuations occur in the A-loop of B-Raf and the P-rich loop of MEK1.


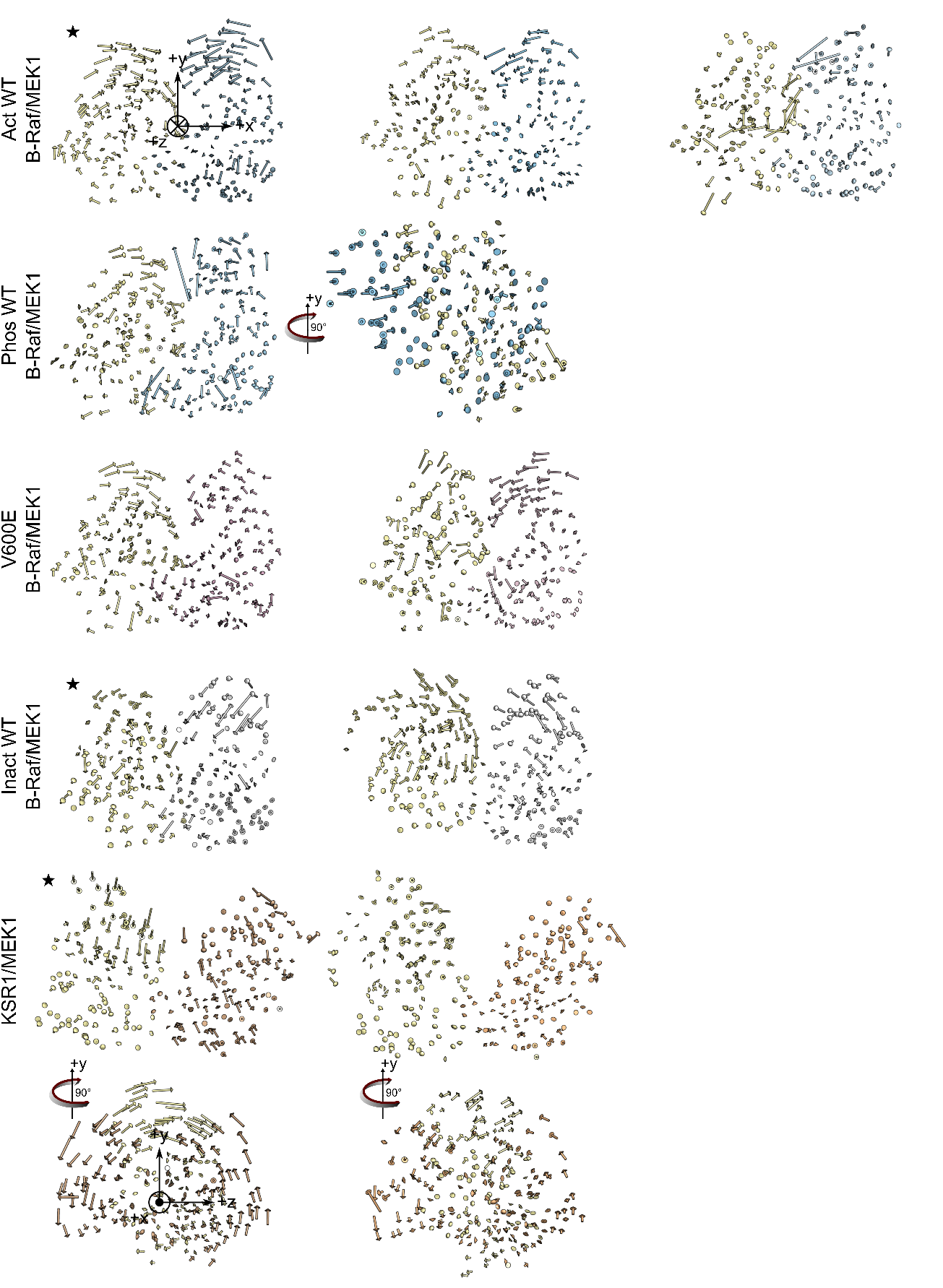
**Fig. S5** The first normal mode for each simulation trajectory shows that B-Raf/MEK1 heterodimers principal motion is to rotate the N-lobes towards the dimer interface, while KSR1/MEK1 heterodimers rotate perpendicular to the dimer interface. Results from trajectories marked with a star are included in the main article.


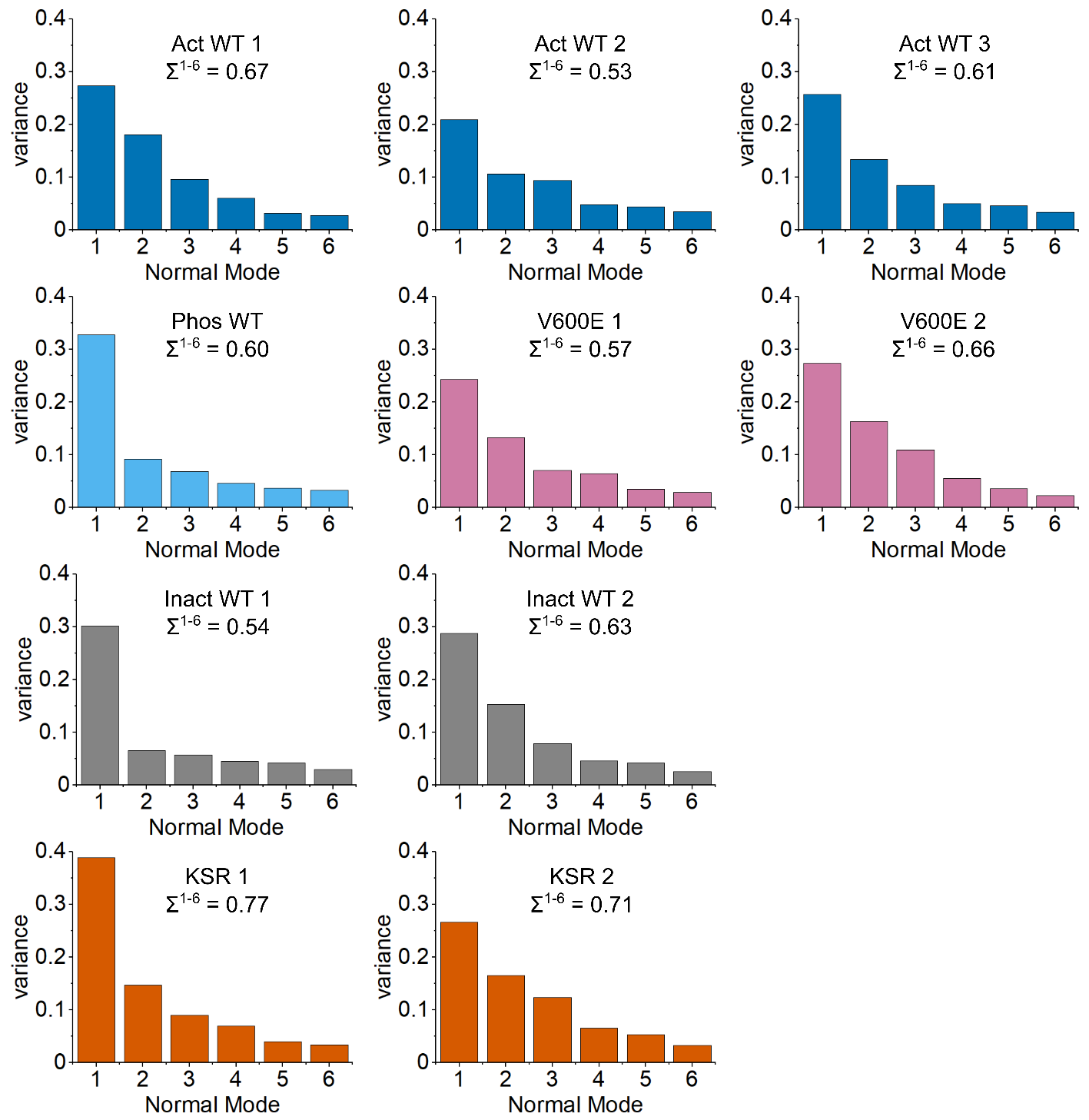
**Fig. S6** Variance of the first six normal modes for each simulation trajectory. The sum of the variance of the first six normal modes is indicated.

**Fig**
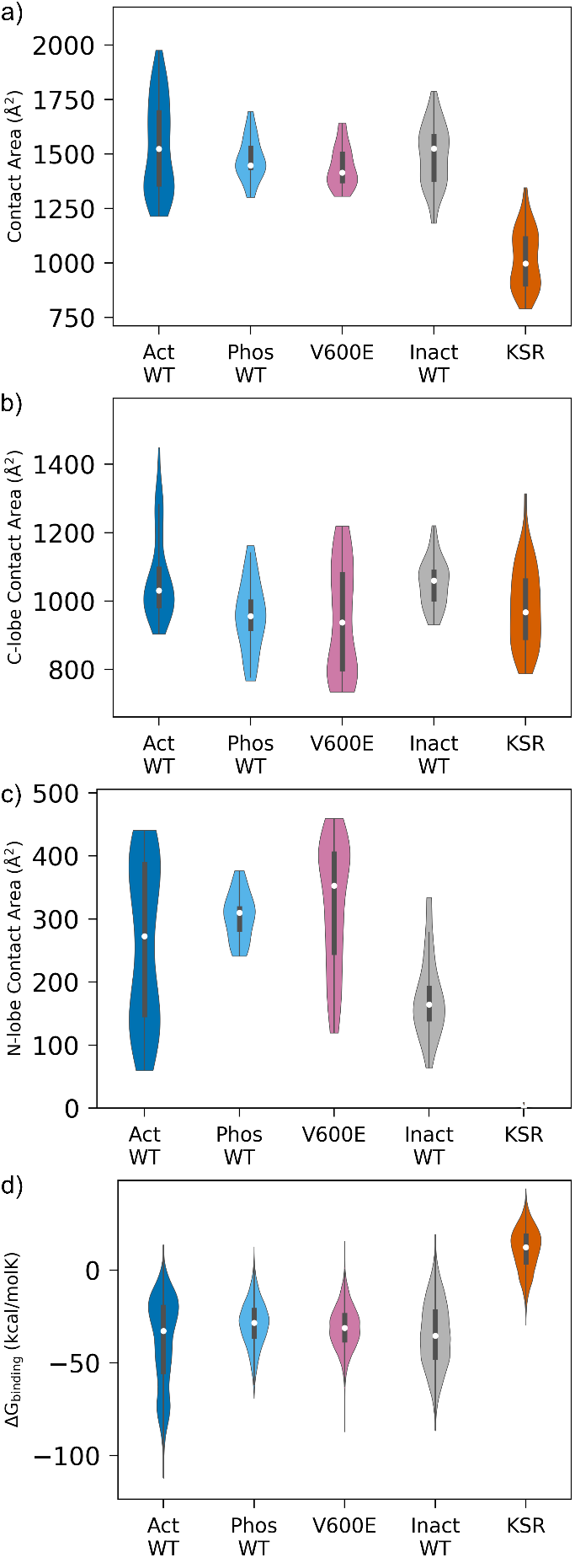
**. S7** Contact area and binding free energy violin-plots for each system show differences in binding between B-Raf/MEK1 and KSR1/MEK1 systems. (a) Total contact area between B-Raf and MEK1 is approximately equal for all B-Raf/MEK1 systems. B-Raf/MEK1 total contact area is greater than KSR1/MEK1 contact area. (b) C-lobe to C-lobe contact area is approximately equal for all systems. (c) N-lobe to N-lobe contact area is slightly higher for phosphorylated B-Raf/MEK1 and B-Raf V600E/MEK1 systems than for inactive B-Raf/MEK1 systems. KSR1/MEK1 system has no N-lobe to N-lobe contact. (d) Binding free energy is equal for B-Raf/MEK1 systems. The value of binding free energy of KSR1/MEK1 is higher than that of the B-Raf/MEK1 systems. In these and similar violin plots, the colored area shows the probability density of the data, the white dot represents the median, and the thick line extending from the median represents the interquartile range (IQR) between the first and third quartile (Q_1_ and Q_3_, respectively). The thin line represents Q_1_−1.5×IQR and Q_3_+1.5×IQR (1.5×IQR range). Data outside the 1.5×IQR range are considered outliers.


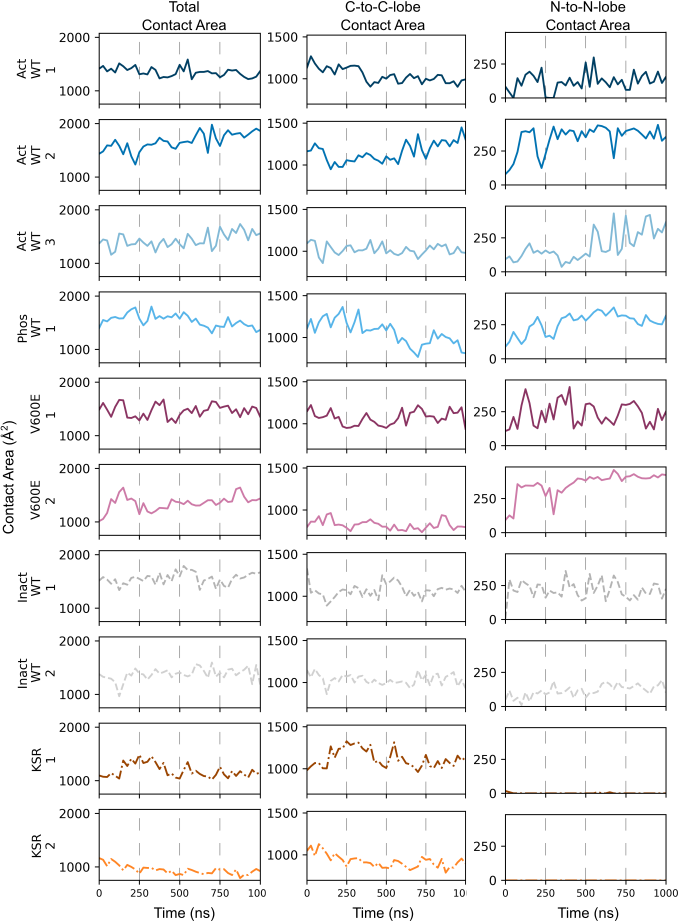
**Fig. S8** There is low correlation between the interfacial contact area of B-Raf/MEK1 (or KSR1/MEK1) and time for each individual trajectory.


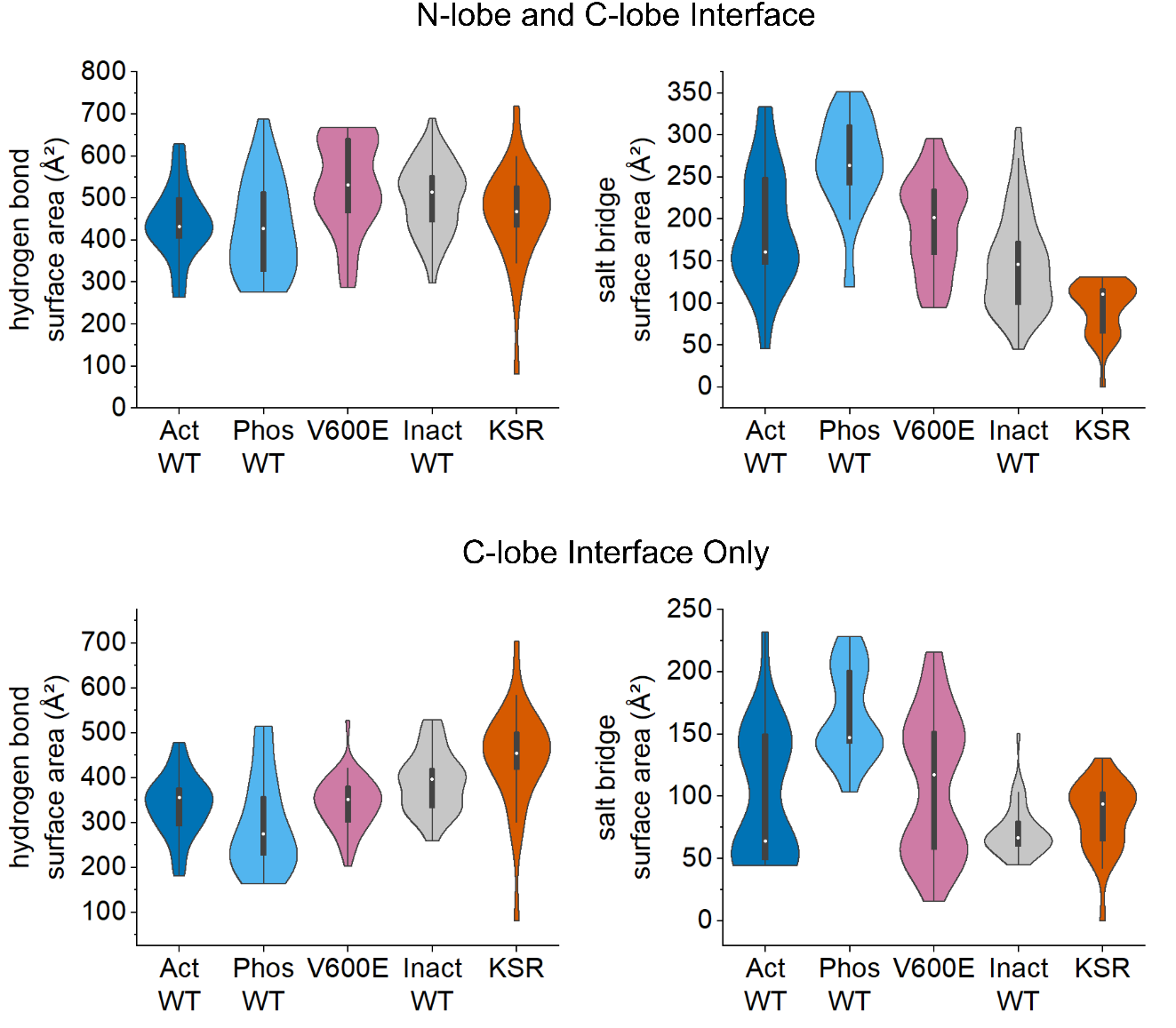
**Fig. S9** Hydrogen bond and salt bridge contributions to the B-Raf/MEK1 and KSR1/MEK1 interfaces. The top plots show the contact surface area of the whole surface (N-lobe + C-lobe interface). The bottom plots show the results when just the C-lobe is considered.


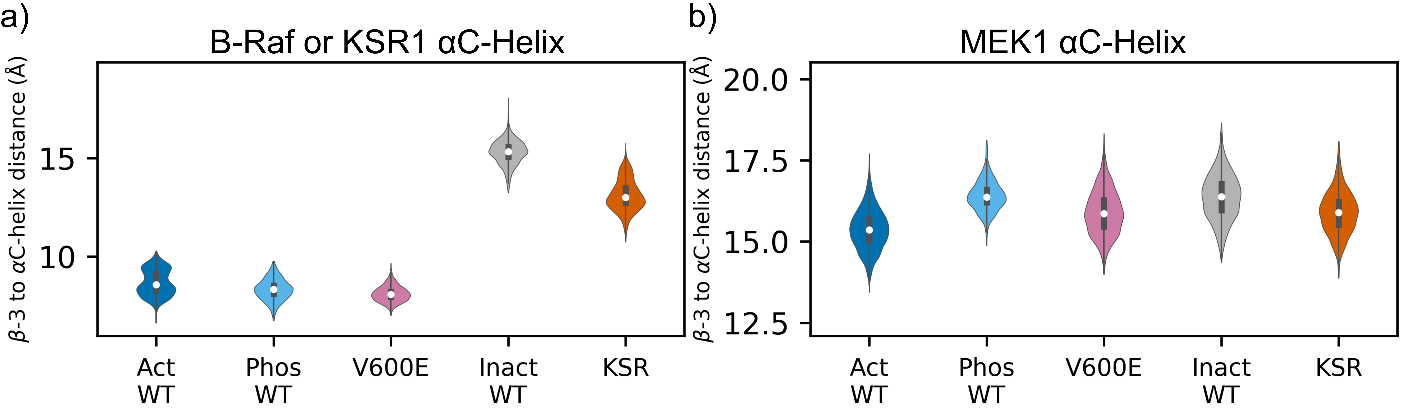


**Fig. S10** (a) Systems which we have designated as having an active B-Raf (active wild-type B-Raf (Act WT), pThr599/pSer602 wild-type B-Raf (Phos WT), and B-Raf V600E (V600E)) have an inward αC-helix. Systems which we have designated as having an inactive B-Raf (Inactive wild-type B-Raf (Inact WT)) as well as the KSR1 system have an outward αC-helix. (b) MEK1 has an outward αC-helix for all systems. The position of the αC-helix is measured as the distance between key residues on β3-strand and αC-helix as described in the main article.


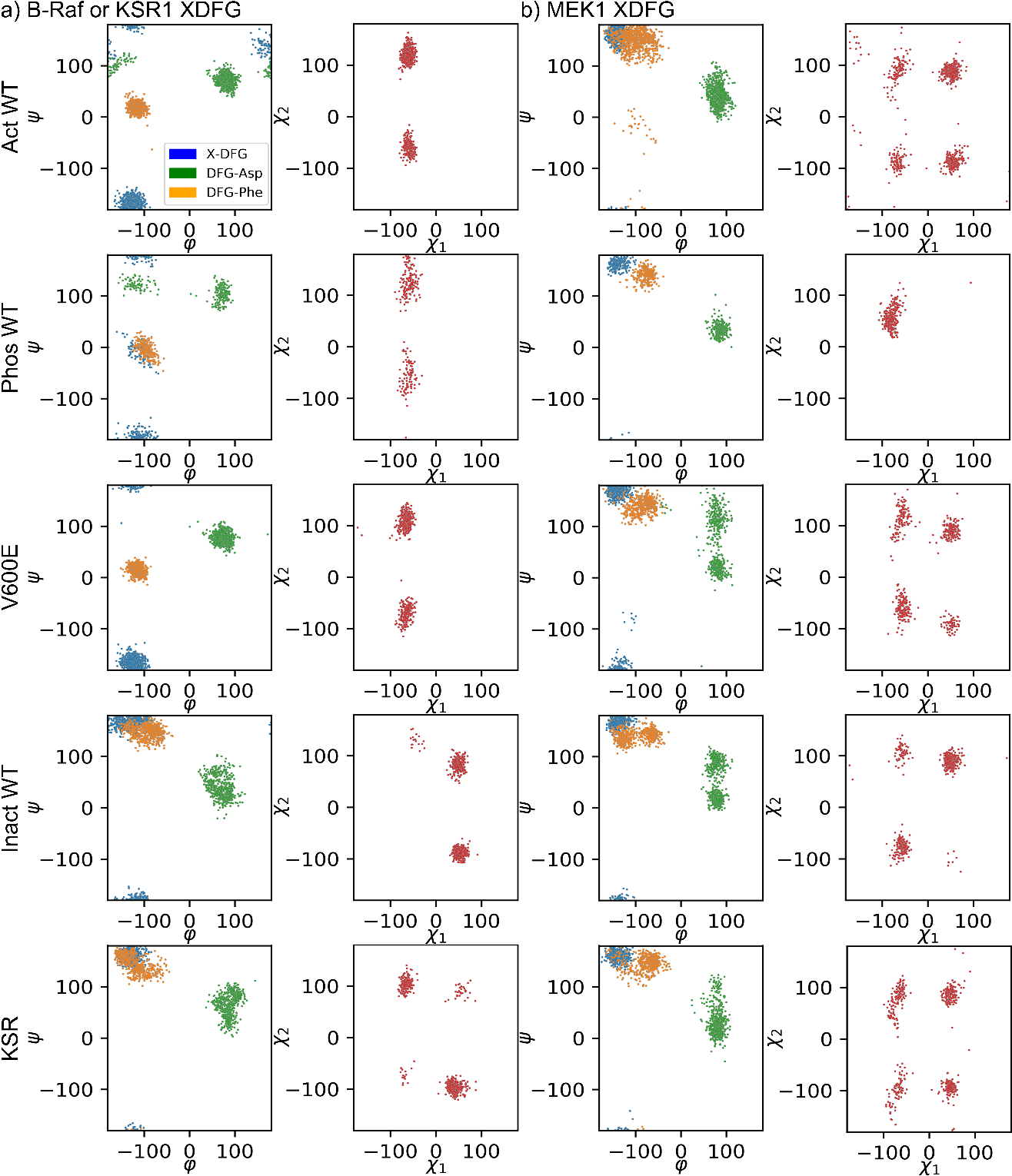
**Fig. S11** (a) Systems which contain active B-Raf (Act WT, Phos WT, V600E) exhibit active orientations of the XDFG motif. Systems which contain inactive B-Raf or KSR1 do not exhibit active orientations of the XDFG motif. (b) MEK1 does not exhibit an active orientation of the XDFG motif.


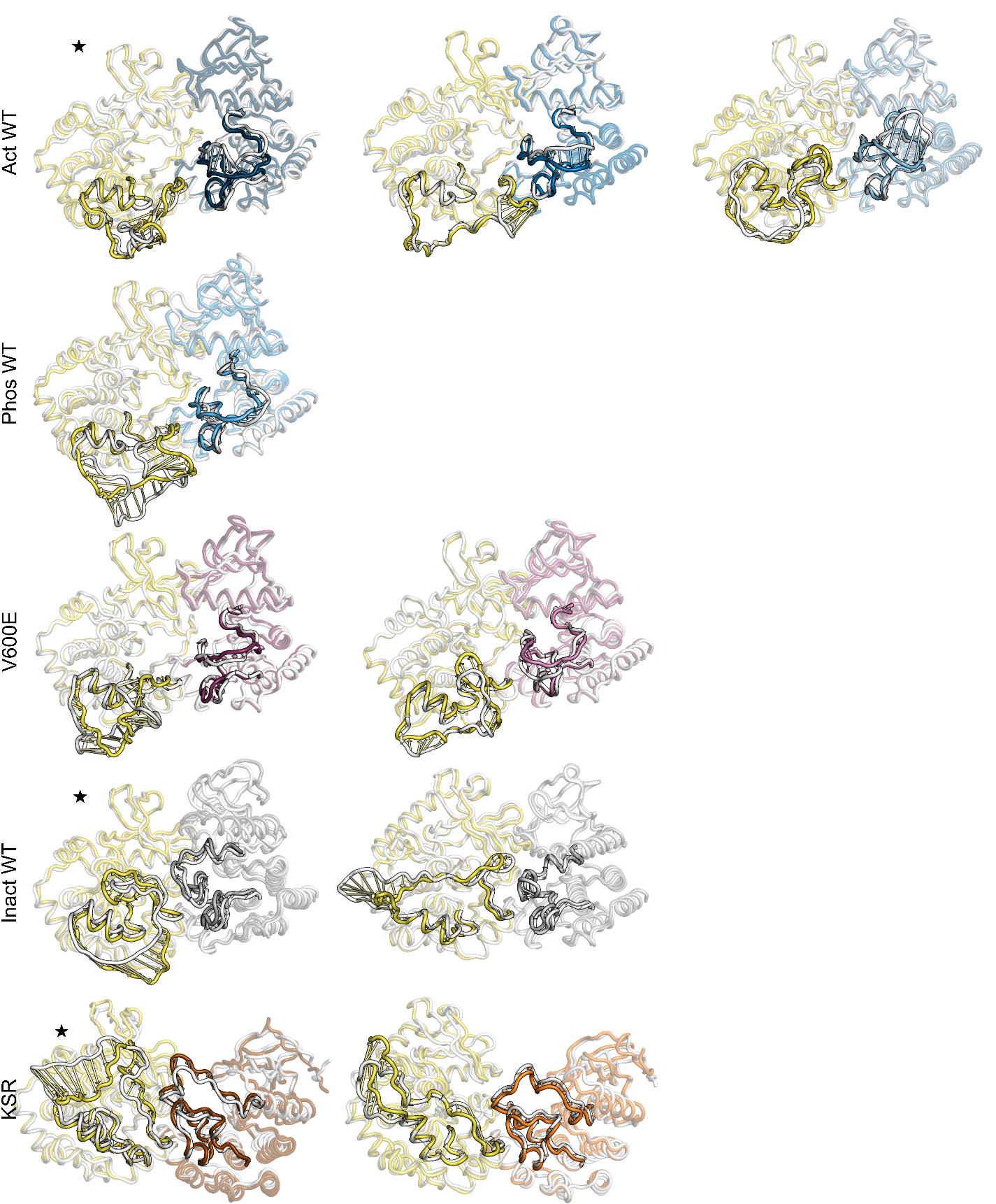
**Fig. S12** The first normal mode of the P-rich loop of MEK1 shows that this loop moves to stay in contact with the A-loop and loop between APE motif and αF-helix of B-Raf/KSR1. Results from trajectories marked with a star are included in the main article.


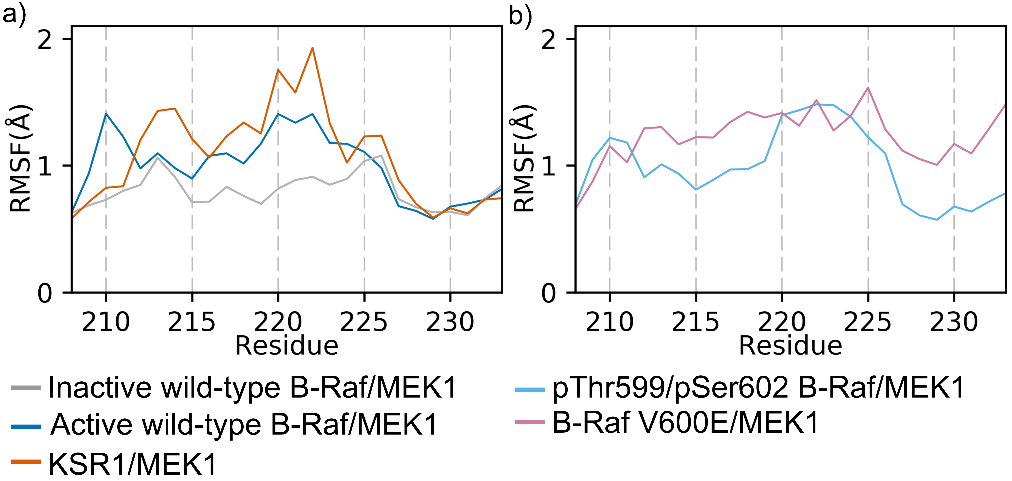


**Fig. S13** (a) MEK1 A-loop (residues 208-227) RMSF values are low in the inactive wild-type B-Raf/MEK1, but higher in the active wild-type B-Raf/MEK1 and KSR1/MEK1 systems. (b) MEK1 A-loop RMSF values are also higher in pThr599/pSer602 wild-type B-Raf/MEK1 and B-Raf V600E/MEK1 systems around the MEK1 serine residues to be phosphorylated (Ser218 and Ser222) than in the inactive wild-type B-Raf/MEK1 system.


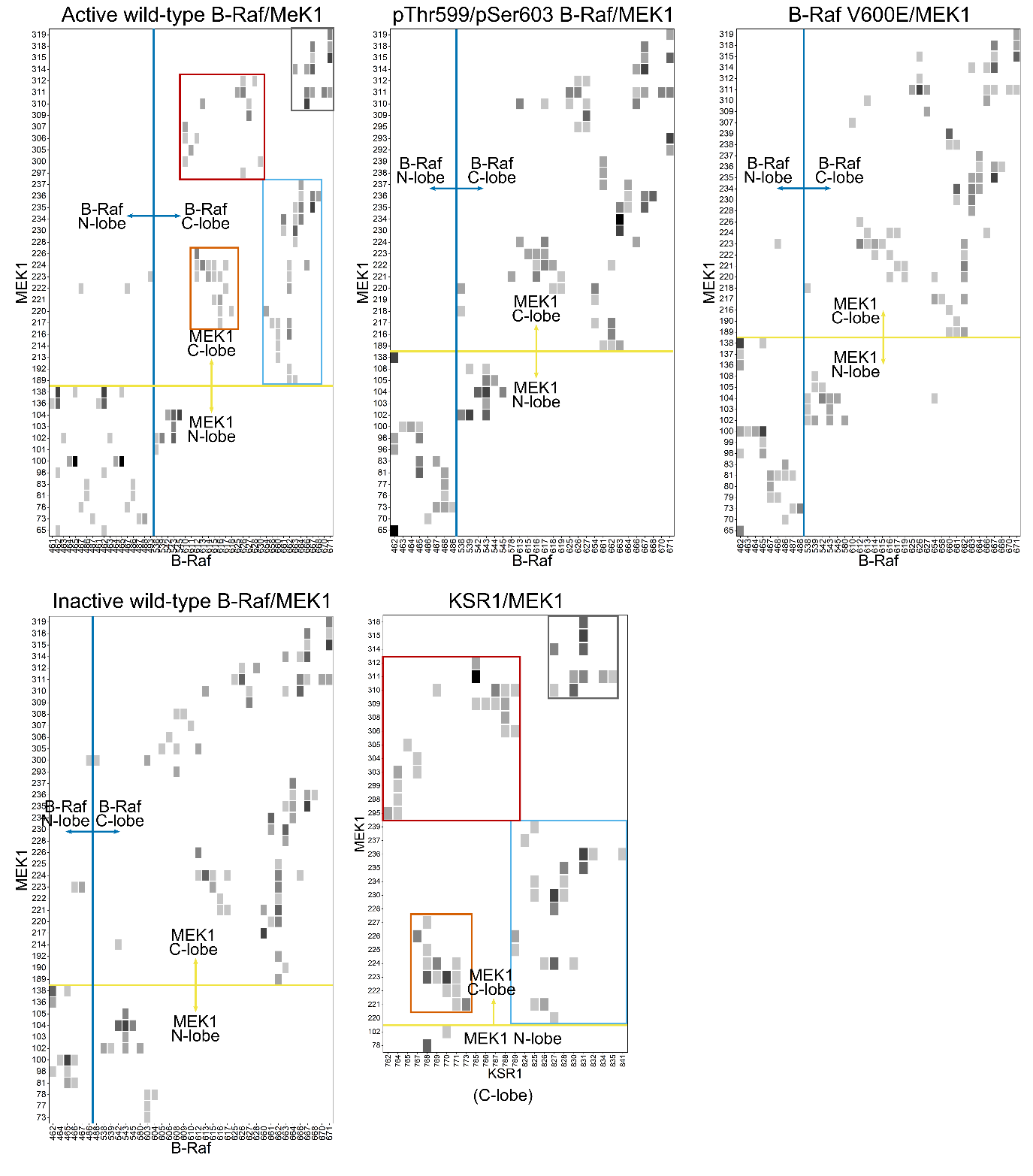
**Fig. S14** Contact maps for all MEK1 and B-Raf (or KSR1) residues that make contact for each system investigated. Contacts are clustered into four regions in all systems: MEK1 A-loop to B-Raf (or KSR1) A-loop (orange box), MEK1 P-rich loop and αG-helix to B-Raf (or KSR1) A-loop through αF-helix (red box), MEK1 αG-helix to B-Raf (or KSR1) αG-helix (gray box), and MEK1 A-loop through αF-helix to B-Raf (or KSR1) αG-helix (blue box). B-Raf/MEK1 systems also exhibit contacts between the N-lobes of the two proteins and between MEK1 β3-stand to αC-helix loop and B-Raf hinge region to αD-helix. Results are scaled by the maximum residue-residue contact area for all systems. Dark colors indicate high residue-residue contact probability, while light colors indicate low residue-residue contact probability. White areas indicate residues that do not interact.


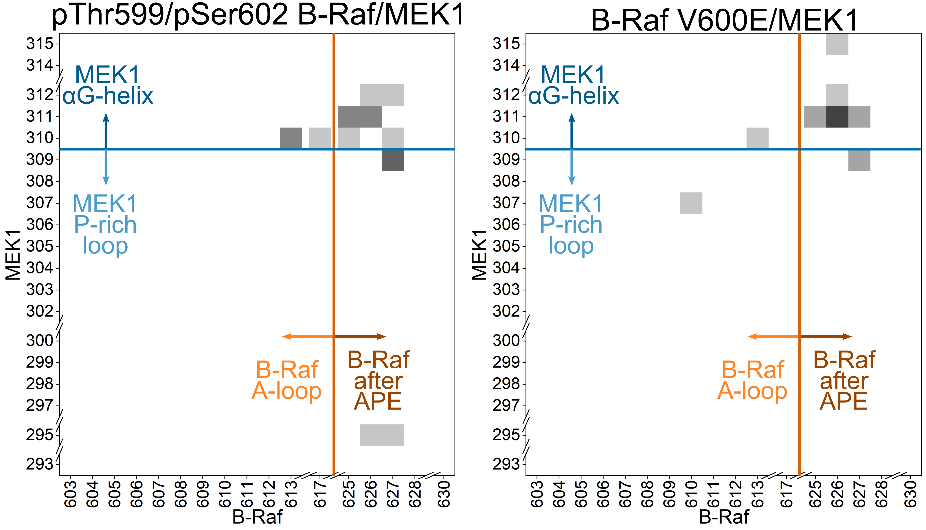


**Fig. S15** Contact maps for MEK1 P-rich loop and αG-helix resides versus B-Raf A-loop residues and the residues between the APE motif and αF-helix for the pThr599/pSer602 B-Raf/MEK1 (left panel) and B-Raf V600E/MEK1 (right panel) systems.


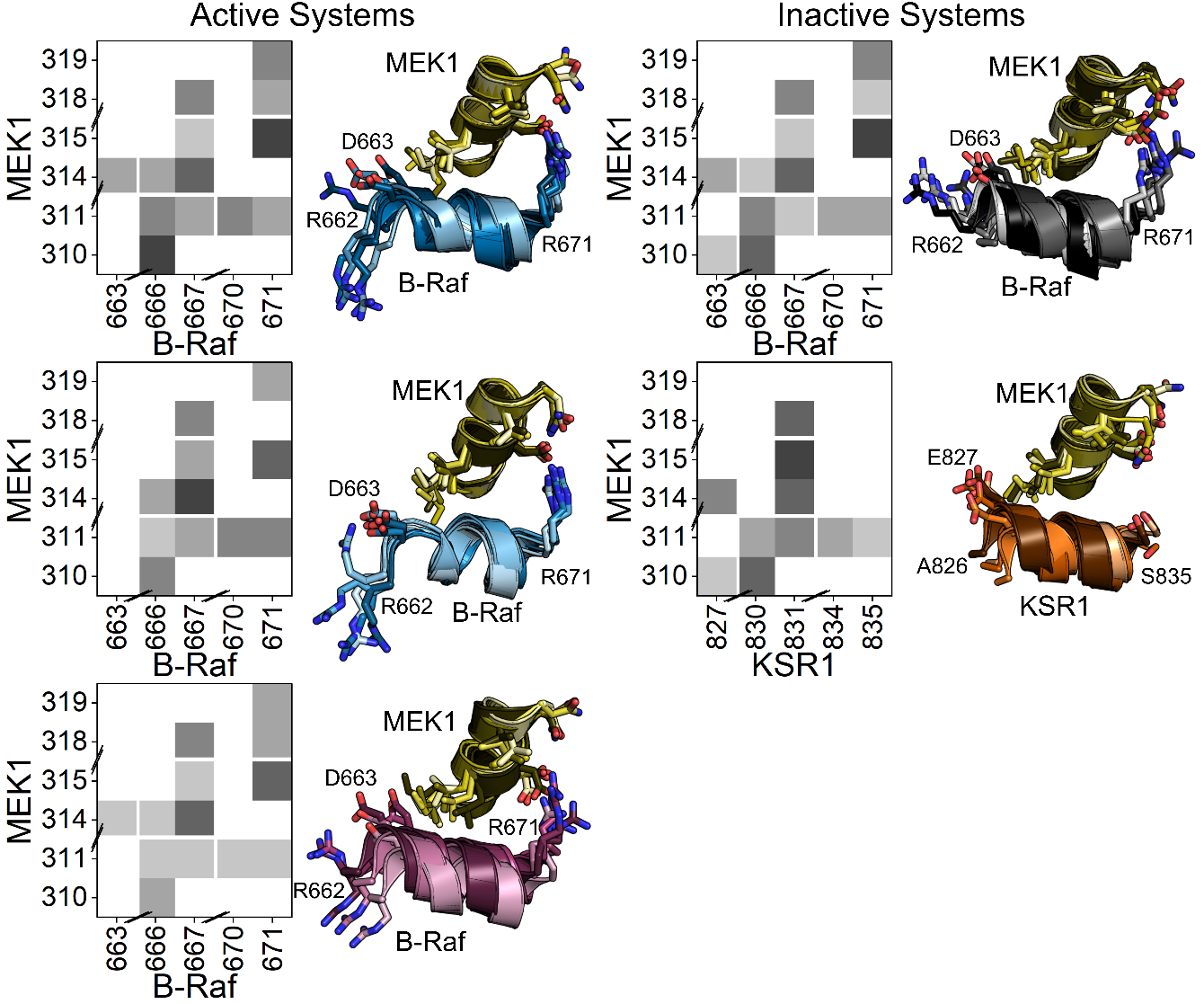


**Fig. S16** Contact maps and representative snapshots for active wild-type B-Raf (top left), pThr599/Ser602 wild-type B-Raf (middle left), B-Raf V600E (bottom left), inactive wild-type B-Raf (top right), and KSR1 (bottom right) systems. More contacts between inactive B-Raf (or KSR1) αG-helix and MEK1 αG-helix can be observed than that for the active B-Raf systems. Darker shades in the color map indicate greater contact area.


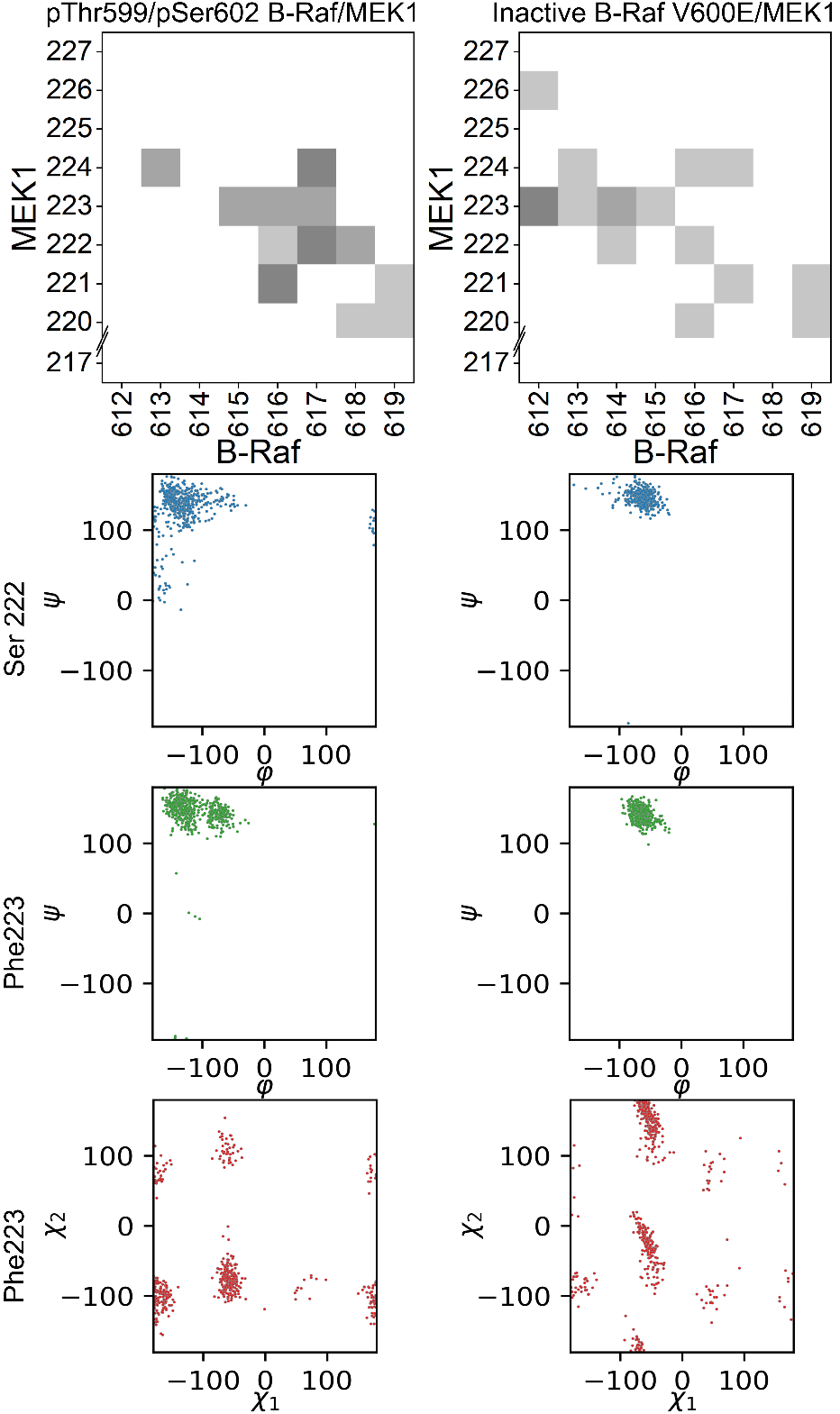
**Fig. S17** Active B-Raf systems exhibit greater flexibility in the MEK1 A-loop. Contact map (top), main chain dihedral angles of Ser222 (blue), main chain dihedral angle of Phe223 (green), and sidechain dihedral angle of Phe223 (red) of MEK1 for pThr599/pSer602 wild-type B-Raf and B-Raf V600E systems.

**Fig. S18** Sequence alignment of the A-loop of MEK1 and MEK2 for various organisms.

MEK1_DICDI 432 - DFGVSGQLQHTLSKAVTWVGTVTYMSPE - 459

MEK1_XENLA 208 - DFGVSGQLIDSM--ANSFVGTRSYMSPE - 233

MEK2_XENLA 294 - DFGISGQLVDSIAKT-RDAGCRPYMAPE - 320

MEK1_CAEEL 211 - DFGIAGRLIESRAHS-KQAGCPLYMGPE - 237

MEK2_CAEEL 213 - DFGVSGMLIDSM--ANSFVGTRSYMAPE - 238

MEK1_HUMAN 208 - DFGVSGQLIDSM--ANSFVGTRSYMSPE - 233

MEK2_HUMAN 212 - DFGVSGQLIDSM--ANSFVGTRSYMAPE - 237

MEK1_MOUSE 208 - DFGVSGQLIDSM--ANSFVGTRSYMSPE - 233

MEK2_MOUSE 212 - DFGVSGQLIDSM--ANSFVGTRSYMSPE - 237

MEK1_RAT 208 - DFGVSGQLIDSM--ANSFVGTRSYMSPE - 233

MEK2_RAT 212 - DFGVSGQLIDSM--ANSFVGTRSYMSPE - 237

MEK1_RABIT 208 - DFGVSGQLIDSM--ANSFVGTRSYMSPE - 233

MEK1_CRIGR 208 - DFGVSGQLIDSM--ANSFVGTRSYMSPE - 226

MEK1_PANTR 208 - DFGVSGQLIDSM--ANSFVGTRSYMSPE - 233

MEK1_SERCA 201 - DFGVSGQLIDSM--ANSFVGTRSYMSPE - 230

MEK2_CHICK 210 - DFGVSGQLIDSM--ANSFVGTRSYMSPE - 235

MEK2_CANLF 212 - DFGVSGQLIDSM--ANSFVGTRSYMSPE - 237

MEK2_CYPCA 209 - DFGVSGQLIDSM--ANSFVGTRSYMSPE – 234


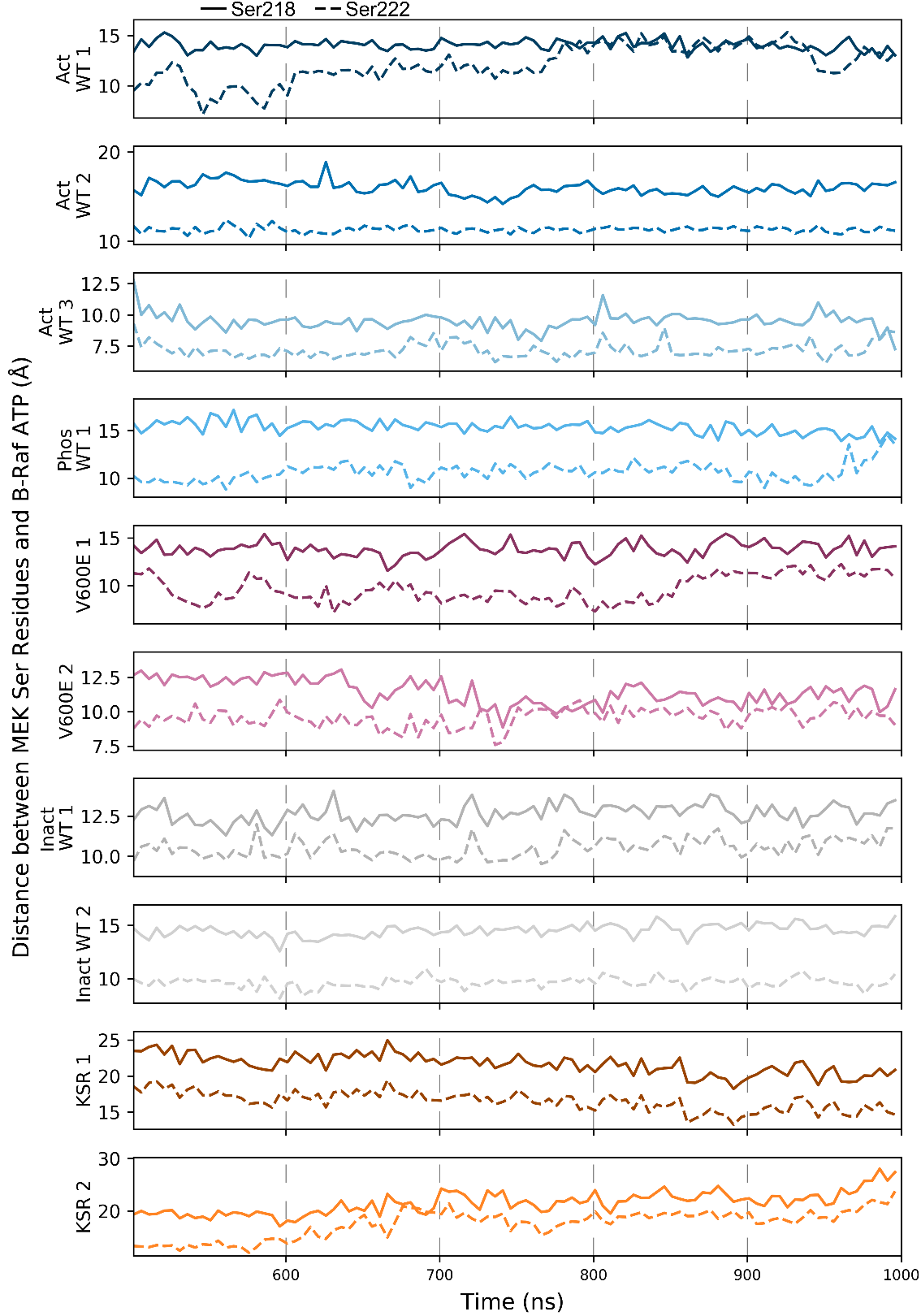


**Fig. S19** Ser222 is generally closer to ATP than Ser218 throughout the simulations; however, if B-Raf is active, Ser218 can approach ATP as well, though with less frequency.


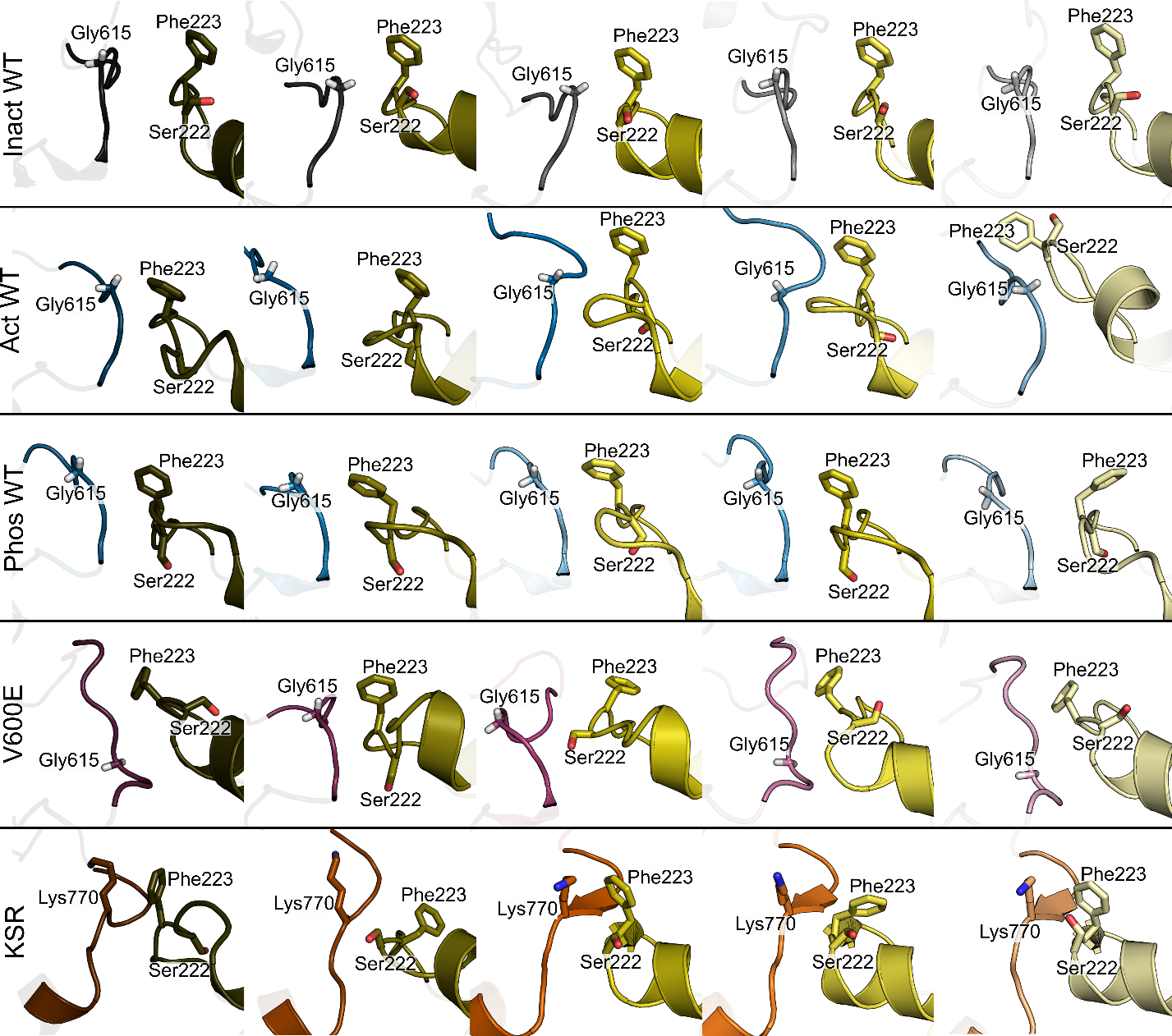
**Fig. S20** Orientations of MEK1 Ser222 and Phe223 do not change greatly for inactive B-Raf/MEK1 systems but can change for active B-Raf/MEK1 and KSR1/MEK1 systems. B-Raf Gly615 and KSR1 Lys770 are shown to give an indication of location of the B-Raf and KSR1 A-loops relative to the MEK1 A-loop.
